# A Self‐Assembled 3D Model Demonstrates How Stiffness Educates Tumor Cell Phenotypes and Therapy Resistance in Pancreatic Cancer

**DOI:** 10.1002/adhm.202301941

**Published:** 2024-03-22

**Authors:** Ying Liu, Babatunde O. Okesola, David Osuna de la Peña, Weiqi Li, Meng‐Lay Lin, Sara Trabulo, Marianthi Tatari, Rita T. Lawlor, Aldo Scarpa, Wen Wang, Martin Knight, Daniela Loessner, Christopher Heeschen, Alvaro Mata, Oliver M. T. Pearce

**Affiliations:** ^1^ Barts Cancer Institute Queen Mary University of London London EC1M 6BQ UK; ^2^ School of Engineering and Materials Science Queen Mary University of London London E1 4NS UK; ^3^ School of Life Sciences Faculty of Medicine and Health Sciences University of Nottingham Nottingham NG7 2RD UK; ^4^ Department of Diagnostics and Public Health Section of Pathology University of Verona Verona 37134 Italy; ^5^ ARC‐Net Applied Research on Cancer Centre University of Verona Verona 37134 Italy; ^6^ Centre for Bioengineering School of Engineering and Materials Science Queen Mary University of London London E1 4NS UK; ^7^ Centre for Predictive in vitro Models Queen Mary University of London London E1 4NS UK; ^8^ Department of Chemical and Biological Engineering Faculty of Engineering Monash University Melbourne VIC 3800 Australia; ^9^ Department of Materials Science and Engineering Faculty of Engineering Monash University Melbourne VIC 3800 Australia; ^10^ Department of Anatomy and Developmental Biology Faculty of Medicine Nursing and Health Sciences Monash University Melbourne VIC 3800 Australia; ^11^ Pancreatic Cancer Heterogeneity Candiolo Cancer Institute – FPO – IRCCS Candiolo (TO) 10060 Italy; ^12^ School of Pharmacy University of Nottingham University Park Nottingham NG7 2RD UK; ^13^ Biodiscovery Institute University of Nottingham University Park Nottingham NG7 2RD UK; ^14^ Department of Chemical and Environmental Engineering University of Nottingham University Park Nottingham NG7 2RD UK

**Keywords:** 3D disease model, drug resistance, pancreatic cancer, peptide amphiphile, self‐assembling hydrogels, tumor microenvironment, tunable stiffness

## Abstract

Pancreatic ductal adenocarcinoma (PDAC) is characterized by a dense and stiff extracellular matrix (ECM) associated with tumor progression and therapy resistance. To further the understanding of how stiffening of the tumor microenvironment (TME) contributes to aggressiveness, a three‐dimensional (3D) self‐assembling hydrogel disease model is developed based on peptide amphiphiles (PAs, **PA‐E3Y**) designed to tailor stiffness. The model displays nanofibrous architectures reminiscent of native TME and enables the study of the invasive behavior of PDAC cells. Enhanced tuneability of stiffness is demonstrated by interacting thermally annealed aqueous solutions of **PA‐E3Y (PA‐E3Y_h_)** with divalent cations to create hydrogels with mechanical properties and ultrastructure similar to native tumor ECM. It is shown that stiffening of **PA‐E3Y_h_** hydrogels to levels found in PDAC induces ECM deposition, promotes epithelial‐to‐mesenchymal transition (EMT), enriches CD133^+^/CXCR4^+^ cancer stem cells (CSCs), and subsequently enhances drug resistance. The findings reveal how a stiff 3D environment renders PDAC cells more aggressive and therefore more faithfully recapitulates in vivo tumors.

## Graphical abstract

A 3D self‐assembled peptide amphiphile hydrogel successfully mimics the nanoarchitecture and mechanical characteristics of pancreatic cancer. This hydrogel is particularly tailored for research involving patient‐derived cells, offering superior adjustability. Notably, hydrogels with increased stiffness selectively enrich for cancer stem cells with heightened invasive capabilities and chemoresistance. Therefore, this platform facilitates detailed analyses of cancer cells, including rare cancer stem cells.

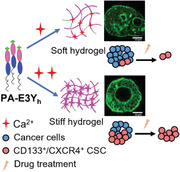

## 1 Introduction

Pancreatic ductal adenocarcinoma (PDAC) is the most common and deadly type of pancreatic cancer with 5‐year survival rate less than 10%.^[^ ^1^ ^]^ The poor treatment response of PDAC is associated with its abundant fibrotic stroma,^[^ ^2^ ^]^ which comprises a dense extracellular matrix (ECM) deposited and crosslinked by both fibroblasts and cancer cells.^[^ ^3^ ^]^ Such stiff ECM not only acts as a physical barrier, limiting the distribution of anti‐cancer drugs to PDAC cells, but also drives aggressive phenotypes such as epithelial‐to‐mesenchymal transition (EMT)^[^ ^4^ ^]^ and cancer stem cells (CSCs) which contribute to metastasis^[^ ^5^ ^]^ and chemoresistance.^[^ ^3^, ^6^ ^]^ To understand these processes mechanistically, suitable three‐dimensional (3D) models are required for the in vitro study of PDAC‐ECM dynamics in the context of drug resistance.^[^ ^7^ ^]^

Most studies of patient‐derived pancreatic cancer samples employ substrates which are either too stiff (>1 GPa, e.g. 2D polystyrene),^[^ ^8^ ^]^ too soft (<1  kPa, e.g. Matrigel),^[^ ^9^ ^]^ or too reliant on a single bioactive molecule (e.g., collagen,^[^ ^10^ ^]^ hyaluronan^[^ ^9^, ^11^ ^]^). Consequently, these substrates can bias cellular responses, make it challenging to study the particular effects of biophysical cues and are poorly predictive of drug response.^[^ ^12^ ^]^ Consequently, it is of utmost importance to dissociate the mechanical attributes of the biomaterial from its chemical composition. Polyacrylamide has been prevalently utilized for tailoring substrate stiffness. However, its application in 3D contexts has often been impeded due to the toxicity of its monomers.^[^ ^13^ ^]^ Recent advancements have mitigated this issue by implementing strategies such as coating the gels with collagen and employing co‐culture techniques with fibroblasts surrounding cancer cells.^[^ ^14^ ^]^ Another commonly used biomaterial, gelatin‐methacryloyl, requires photopolymerization for stiffening, which can be toxic,^[^ ^15^ ^]^ while alginate hydrogels require the simple addition of divalent cations (Ca^2+^) to tune stiffness, but do not resemble the ECM of human tissues.^[^ ^13^ ^]^ In contrast, peptide‐based hydrogels offer the opportunity to customize stiffness while recapitulating the fibrous architecture of the TME. Peptide amphiphiles (PAs)^[^ ^16^ ^]^ are a class of self‐assembling peptides that spontaneously organize into nanofibrous hydrogels while displaying specific bioactive epitopes on the surface. These amphiphilic molecules assemble into well‐defined nanofibers through hydrophobic interactions from their hydrophobic palmitoyl tail and hydrogel bonding triggered upon charge screening. We have taken advantage of multicomponent self‐assembly^[^ ^17^ ^]^ to enhance control of structural^[^ ^18^ ^]^ and signaling^[^ ^19^ ^]^ properties of PA‐based hydrogels. In particular, we demonstrated how co‐assemblies of PAs and specific ECM molecules present in ovarian^[^ ^20^ ^]^ and pancreatic^[^ ^19^ ^]^ cancer are used as 3D disease models, recapitulating structural and compositional features of the TME. We showed that these PA‐based platforms can support the growth of pancreatic CSCs and capture in vivo drug resistance.

CSCs, also described as tumor‐initiating cells, are a subset of cancer cells with unlimited self‐renewal capacity and the ability to initiate and sustain tumor growth.^[^ ^21^ ^]^ Despite their relative sparsity, these cells play a major role in chemotherapeutic resistance, cancer metastasis, and tumor relapse,^[^ ^22^ ^]^ thereby markedly affecting patient outcome. Moreover, increasing evidence suggests that CSC populations are dynamic and fluctuate between the non‐CSC to CSC states through crosstalk with their microenvironment.^[^ ^23^ ^]^ In particular, stiffening of the TME has been shown to regulate CSC self‐renewal and migration and to induce EMT through sustained mechanotransduction via YAP or Rho/ROCK.^[^ ^24^ ^]^ Therapeutic targeting of these pathways has corroborated their contribution to PDAC's poor survival. For example, transient tissue softening with the ROCK inhibitor fasudil primes PDAC tumors to respond to chemotherapy, significantly reducing invasion and metastasis in mice.^[^ ^6^ ^]^ Similar results have been obtained by targeting ECM crosslinkers such as lysyl oxidase (LOX)^[^ ^25^ ^]^ as well as the master mechanoregulator FAK.^[^ ^26^ ^]^ Another approach is to target stiff tissue areas with engineered mechanosensitive mesenchymal stem cells, which are capable of locally activating systemic chemotherapy to eliminate metastases.^[^ ^27^ ^]^ The aforementioned cases underscore the significant translational prospects of targeting tissue stiffness within the realm of oncology, as demonstrated by the perseverance despite the failure of several methods, such as hyaluronan degradation, in clinical trials.^[^ ^28^ ^]^ To fully comprehend these setbacks, a detailed exploration into the biological effects of tissue stiffening on cancer cells is crucial. Such exploration requires platforms that support complex co‐cultures and sophisticated assays, a need made more pressing in the wake of recent successes in concurrent stromal and immune targeting.^[^ ^29^ ^]^

Here, we report on the design of PA‐based hydrogels capable of displaying a spectrum of stiffness, spanning several orders of magnitude reminiscent of values observed for PDAC patient‐derived xenograft (PDX) tissues (1.7 to 11.5 kPa). We have assayed the acquisition of CSC and EMT phenotypes in patient‐derived PDAC cells grown in PA‐based hydrogels and looked at their association with chemotherapeutic resistance in response to increased matrix stiffness.

## 2 Results and Discussion

### 2.1 Rationale for the Material Design

**Figure 1:**
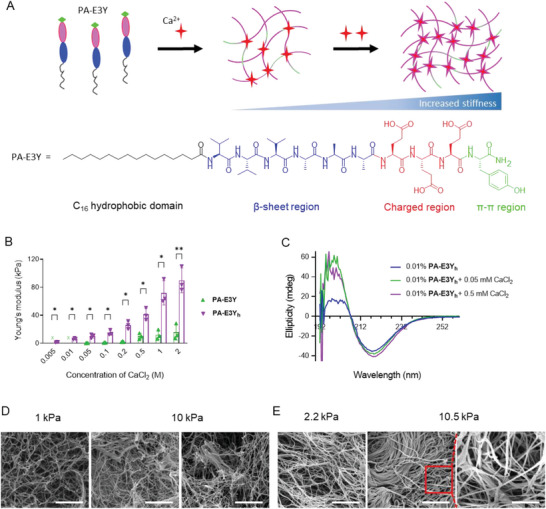
Tunable stiffness and conformation of **PA‐E3Y** using divalent ions. A) Schematic of calcium ion crosslinking of the PA‐E3Y_h_ nanofibers with increasing Ca^2+^ ions, shown as red stars. The sequence of the **PA‐E3Y** molecule shows its structural domains. B) Stiffness of **PA‐E3Y** (unheated) and **PA‐E3Y**_**h**_ (pre‐heated) hydrogels according to CaCl_2_ concentration. C) Circular dichroism spectra of **PA‐E3Y**_**h**_ with and without CaCl_2_. D) Nanoscale morphology of soft and stiff **PA‐E3Y**_**h**_ hydrogels as imaged by SEM (scale bar: 1 µm). E) The nanofibrous architecture of the decellularized soft and stiff PDAC PDX tissue as images by SEM (scale bar: 2 µm, inset scale bar: 1 µm). *P < 0.05; **P < 0.01; ***P < 0.001; ****P < 0.0001.

The stiffness of PDAC stroma (>10 kPa)^[^ ^30^ ^]^ is several orders of magnitude higher than healthy pancreas (≤1 kPa).^[^ ^3^, ^31^ ^]^ We hypothesized that an in vitro PDAC model that is based on hydrogels with tunable stiffness spanning the stiffness landscape of PDAC stroma and healthy pancreas will aid in better reproducing the crosstalk between matrix stiffness, invasive potential, and resistance to chemotherapeutics. Therefore, we developed a PA molecule to produce hydrogels with tunable structural properties. The **PA‐E3Y** (C_16_‐V3A3E3Y) molecule was designed to assemble into nanofibers through the classical PA‐assembly mechanism and included three carboxylic acid moieties to bind calcium (Ca^2+^) ions and a terminal tyrosine (Y) residue to provide π–π stacking. Different PA molecules are distinguished according to their epitope sequence, for example, **PA‐E3Y**. In addition to other multiple non‐covalent interactions (hydrogen bonding provided by the peptide backbone, hydrophobic interactions from the hydrophobic palmitoyl tail, and ionic crosslinking between calcium ions and carboxylate groups), the terminal tyrosine (Y) residue engages in π–π stacking to further enhance the stiffness of **PA‐E3Y** hydrogels. By controlling the stoichiometric ratio of Ca^2+^ ions and **PA‐E3Y** in an aqueous media, it is possible to create hydrogels with a broad spectrum of stiffness which can be further increased if the PAs are transiently pre‐heated (**Figure** **1**A). Also, annealing of the gelator solutions by heat‐cool cycling followed by the addition of Ca^2+^ ions as generated hydrogels with robust mechanical properties as previously demonstrated.^[^ ^32^ ^]^

### 2.2 Ca^2+^‐Induced Gelation of **PA‐E3Y** Hydrogels with Tunable Stiffness

We designed and synthesized **PA‐E3Y** using a previously reported method.^[^ ^33^ ^]^ We used mass spectrometry and analytical high‐performance liquid chromatography (HPLC) to confirm purity of **PA‐E3Y** (Figure S1, Supporting Information). To create hydrogels of **PA‐E3Y** with high stiffness, we pre‐heated an aqueous solution of **PA‐E3Y** (1% wt) to 80 °C and let it cool down to room temperature. Then, we added various concentrations (0.005 to 2.000 M) of CaCl_2_ to the resulting viscous PA solution (**PA‐E3Y_h_**) to assemble hydrogels with tunable stiffness.

To measure the storage (G′) and loss moduli (G″) of the hydrogels as a function of frequency and oscillatory amplitude, we used dynamic oscillatory rheology. The frequency sweep rheographs of unheated **PA‐E3Y** and pre‐heated **PA‐E3Y_h_** hydrogels (1% wt) showed that the moduli (G′ and G″) are independent of oscillatory frequency and that G′ is greater than G″, which is typical of gel‐like materials (Figure S2A, Supporting Information). Furthermore, amplitude sweep rheographs showed that G′ and G″ were relatively constant between 0.1 and 1% strains (Figure S2B, Supporting Information), indicating the linear viscoelastic region (LVR) of the hydrogels, but exhibited non‐linear response beyond the LVR in a similar fashion to natural ECM proteins.^[^ ^34^ ^]^

Using various concentrations of CaCl_2_, we demonstrated the self‐assembly of **PA‐E3Y_h_** hydrogels with a wide range of stiffness. As shown in Figure 1B, the Young's modulus of **PA‐E3Y_h_** hydrogels increased from ≈1 to 90 kPa when the concentration of CaCl_2_ was increased from 0.005 to 2.000 M, while the Young's modulus of unheated **PA‐E3Y** hydrogels increased stiffness from ≈0.35 to 15 kPa only. It is noteworthy that no gelation was observed with the unheated **PA‐E3Y** below 0.05 M CaCl_2_. The utilization of **PA‐E3Y** and **PA‐E3Y_h_** provides a straightforward approach to modulate hydrogel stiffness across a wide range, spanning from 0.35–250 kPa. This can be achieved either by adjusting the Ca^2+^ ion concentration while keeping the gelator concentration constant (Figure 1B) or by altering the gelator concentration while maintaining a constant Ca^2+^ ion concentration (Figure S2C, Supporting Information).

**PA‐E3Y_h_** hydrogels displayed more elastic behavior than Matrigel as evidenced by their lower tan δ values (Figure S2D, Supporting Information). They also exhibited thixotropic properties after the application of high shear loads during a dynamic time‐sweep experiment. Under high shear load, the hydrogels undergo an internal breakage as indicated by the inversion of G″ and G′, which is accompanied by significant decrease and increase in G′ and G″ values, respectively. After three cycles of amplitude sweep, hydrogels of **PA‐E3Y_h_** displayed 90% (± 5.2%) self‐recovery (Figure S2E, Supporting Information), which would enable their use as an injectable material, for example.

To gain insight into the molecular mechanism underpinning Ca^2+^ ion‐induced tuneability of **PA‐E3Y_h_** hydrogel stiffness, we used circular dichroism (CD) spectroscopy to assess a potential conformational change of PAs. As expected, the CD spectra of an aqueous solution of **PA‐E3Y_h_** (0.01% wt) without Ca^2+^ ions at neutral pH depicted both positive and negative bands at 196 and 218 nm, respectively (Figure 1C), which is indicative of a β‐sheet conformation. Upon the addition of CaCl_2_ (0.05 mM), the intensity of CD bands at 196 and 218 nm increased from 18 and 35 mdeg to 55 and 37 mdeg, respectively. Further addition of CaCl_2_ (0.5 mM) increases the intensity of CD band at 218 nm from 35 to 40 mdeg. The heightened intensity of the CD spectra may reflect chirality amplification, which arises due to the restriction of conformational freedom within the self‐assembled **PA‐E3Y_h_**.^[^ ^18^ ^]^

Given the key role of β‐sheet formation in PAs nanofiber assembly, we then assessed the self‐assembly of PAs molecules using Thioflavin T (ThT) staining, a molecular rotor known to become strongly fluorescent upon binding to β‐sheet rich fibrils. Confocal laser scanning microscopy of an aqueous solution of **PA‐E3Y_h_** (0.2% wt) treated with ThT (0.4 mM) in the presence of CaCl_2_ (1 mM) revealed a network of fluorescent ECM‐like nanofibers (Figure S2F, Supporting Information), confirming a strong β‐sheet presence. The nanofibers appeared to be more bundled in the presence of higher concentrations of CaCl_2_ (10 mM), which is consistent with the structural changes revealed by our CD data. Similarly, scanning electron microscopy (SEM) (Figure 1D) of dried **PA‐E3Y_h_** aerogels (1% wt), prepared via a critical point drying process,^[^ ^32b^ ^]^ displayed more bundled nanofibers with stiff hydrogels (10 kPa) prepared with a higher concentration of CaCl_2_ than the soft hydrogels (1 kPa).

The microstructure of the matrix of native tissues affects cell behavior and consequently signaling pathways through cell‐matrix interactions, cell communication, and enrichment of proteins and secreted factors.^[^ ^35^ ^]^ Therefore, the architecture of the hydrogel is an important feature of 3D cell culture platforms aiming to mimic natural tissue. We compared the architecture of the hydrogels against established in vivo PDX tissue that is now widely considered the gold‐standard for translational research in PDAC and other cancer types.^[^ ^36^ ^]^ The morphological discrepancy between 1 and 10 kPa **PA‐E3Y_h_** hydrogels (1% wt) is reminiscent of the soft (≈2.2 kPa) and stiff (10.5 kPa) native tissue derived from PDAC patients (Figure 1E). We observed that both stiff **PA‐E3Y_h_** hydrogels and stiff PDAC PDXs displayed bundled nanofiber networks while the soft **PA‐E3Y_h_** hydrogels and soft tissue showed loose nanofibrous architecture (Figure 1D,E; Figure S2G, Supporting Information). We reasoned that these larger fibers result from bundling of individual nanofibers and underpin the increased stiffness of both stiff **PA‐E3Y_h_** hydrogels and PDAC PDXs, respectively. Taken together, the molecular self‐assembly of **PA‐E3Y_h_** into self‐supporting hydrogels is hierarchical and directed by dynamic non‐covalent interactions (COOˉ—Ca^2+^, π‐Ca^2+^, π‐π, hydrogen bond, and hydrophobic interactions). The Ca^2+^ induced tunable stiffness of **PA‐E3Y_h_** hydrogels suggests a stepwise crosslinking of the nanofibers that resembles the morphological reorganization that mediates progressive TME stiffness in PDAC patients.

### 2.3 Quantification of PDAC Mechanical Properties in Tumor and Stroma

**Figure 2:**
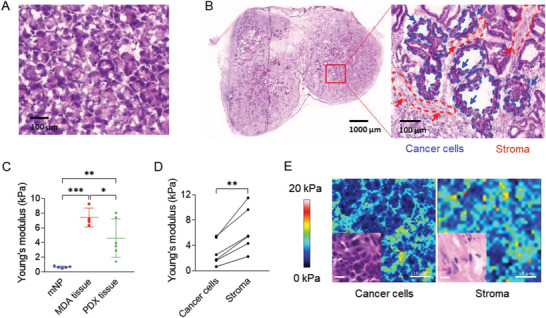
ECM mechanical properties of patient‐ and mouse‐derived pancreatic cancer in vivo models. HE staining of A) mouse normal pancreatic tissue (mNP) and B) PDX tissue was used to select area containing predominantly cancer cells or stromal cells for subsequent AFM measurements (scale bar: A: 100 µm; B: 1000 µm, inset: 100 µm). C) Stiffness of mNP (n = 5), MDAs (n = 4) and PDXs (n = 6) measured by AFM. D) Stiffness of matched cancer and stromal areas in PDX. E) The force map and H&E staining (inset) of cancer and stromal area in PDXs (scale bar in force map and H&E staining: 10 µm). Three frozen tissue sections for each specimen were independently measured by AFM. Five force maps were obtained from each section, and every force map covered a size of 50 × 50 µm^2^ region under 10 × 10 point grids representing 100 force curves. *P < 0.05; **P < 0.01; ***P < 0.001.

To determine the appropriate stiffness range of **PA‐E3Y_h_** hydrogels for PDAC modeling, we quantified the stiffness of pancreatic cancer tissue obtained from six pancreatic cancer PDXs and four mouse‐derived allografts (MDAs) using atomic force microscopy (AFM). We used AFM to measure the rigidity of specific areas of the tissue and used murine normal pancreas (mNP) as a control (**Figure** **2**A). Hematoxylin and eosin (H&E) staining allowed us to distinguish between areas composed of cancer cells and adjacent desmoplastic areas containing almost exclusively stromal cells (Figure 2B). Consecutive cryosections^[^ ^30a^ ^]^ were then used for AFM. The observed stiffness of PDX samples from six patients ranged from 0.5 to 15 kPa, with 92% of the area distributed between 1 and 10 kPa. The stiffness values observed are markedly greater than those recorded for mNP, which stand at 0.5 kPa (Figure 2C). However, they are lower and demonstrate more variability compared to MDAs. This is likely indicative of the intrinsic heterogeneity among PDXs, as well as the differing extents of stromal recapitulation by the murine host. For each PDX sample we compared stiffness values for the stroma (1.7 to 11.5 kPa) with those of the tumor (0.5 to 5.0 kPa), suggesting that the stiffness of stromal areas is approximately twofold higher (Figure 2D,E). A similar pattern is observed for MDA tissues (6 to 13 kPa) (Figure S3A, Supporting Information). Since the PDAC cells used in this study were isolated from PDX tissue, we decided to use **PA‐E3Y_h_** hydrogels with a stiffness range of 1.7 to 11.5 kPa for subsequent experiments.

### 2.4 Hydrogel Stiffness Regulates PDAC Cell Behavior

**Figure 3:**
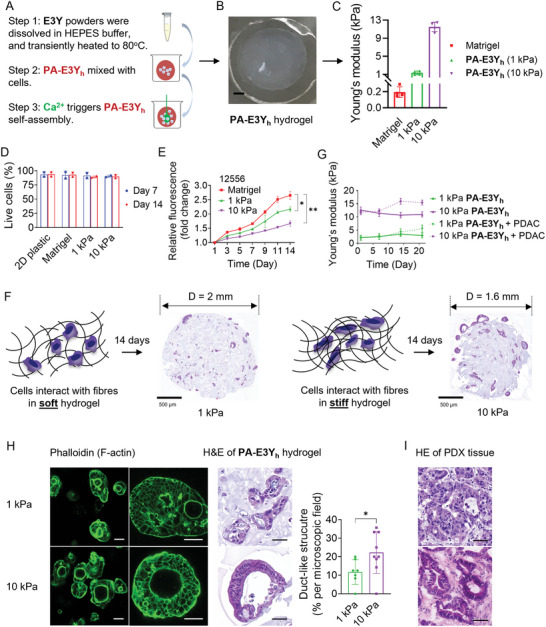
Cell encapsulation in E3Y PA hydrogel with different stiffness. A) Schematic of the process of PDAC cell culture in **PA‐E3Y**_**h**_ hydrogels. B) Representative image of **PA‐E3Y**_**h**_ hydrogels on PDMS substrate (scale bar: 1 mm). C) Stiffness of Matrigel, soft and stiff **PA‐E3Y**_**h**_ as measured by rheometry (*n* = 4). D) Cell viability for PDAC cells in 2D, Matrigel, **PA‐E3Y**_**h**_ hydrogels (1 and 10 kPa) as assessed by flow cytometry using DAPI on day 7 and 14 (*n* = 3). E) Proliferation of PDAC cells (12556) in Matrigel, 1 and 10 kPa **PA‐E3Y**_**h**_ hydrogels as assessed by alamar blue over 14 days. F) Schematic diagram of cells interacting with nanofibers in 1 and 10 kPa **PA‐E3Y**_**h**_ hydrogels (left) with their corresponding H&E stains (right), scale bar: 500 µm. G) Stiffness of **PA‐E3Y**_**h**_ hydrogels with PDAC cells (12556) after 21 days in culture in sphere medium, as measured by rheometry (*n* = 3). H) Formation of PDAC duct‐like structures in **PA‐E3Y**_**h**_ hydrogels as imaged by immunofluorescence and H&E staining. Histogram shows quantification of duct‐like structures in 1 and 10 kPa **PA‐E3Y**_**h**_ hydrogels (*n* = 3). Scale bar on IF images at the left side: 100 µm, the right side: 50 µm; scale bar in HE images: 50 µm. I) H&E staining of PDAC PDX tissue (scale bar: 50 µm). *P < 0.05; **P < 0.01.

The soft (1 kPa) and stiff (10 kPa) hydrogels of **PA‐E3Y_h_** were prepared with 5 mM and 50 mM of CaCl_2_ (the final concentrations of Ca^2+^ are 1.25 mM and 12.5 mM), respectively (**Figure** **3**A–C), to investigate the effects of tumor stroma stiffness on PDAC characteristics. Both 2D tissue culture plastic and Matrigel (0.2 kPa, Figure 3C) were also tested. First, we used flow cytometry to demonstrate that PDAC cells encapsulated in both soft and stiff **PA‐E3Y_h_** hydrogels exhibited high cell viability (> 90%) after 14 days in culture, which is indeed comparable to results for cells embedded in Matrigel or seeded on 2D plastic (Figure 3D; Figure S4A, Supporting Information). Next, we assessed PDAC cell proliferation by measuring resazurin reduction following 14 days of culture in soft and stiff **PA‐E3Y_h_** hydrogels versus Matrigel. PDAC cell numbers progressively increased in **PA‐E3Y_h_** hydrogels (Figure 3E), demonstrating the ability of both soft and stiff **PA‐E3Y_h_** hydrogels to support cell expansion. However, lower cell proliferation was observed in stiff **PA‐E3Y_h_** hydrogels compared to Matrigel and soft **PA‐E3Y_h_** hydrogels, respectively. Notably, cell‐laden stiff **PA‐E3Y_h_** hydrogels shrank by ≈20% (H&E staining of the **PA‐E3Y_h_** hydrogel in Figure 3F) and displayed higher stiffness than the cell‐free stiff **PA‐E3Y_h_** hydrogels after 14 days in culture (Figure 3G). The observed shrinkage of the cell‐laden stiff **PA‐E3Y_h_** hydrogels might be attributed to cellular contraction and matrix remodeling, while matrix stiffening might be due to proliferation of PDAC cells and production of (secreted) matrix‐modulating proteins by the encapsulated cells.^[^ ^34^ ^]^ In addition, the bundled nanofiber networks of **PA‐E3Y_h_** hydrogels could aid in the retention and accumulation of secreted proteins, leading to enhanced hydrogel stiffness as previously reported.^[^ ^37^ ^]^

PDAC cells encapsulated in the hydrogels formed colonies which varied in size and number (Figure S4B, Supporting Information). Stiff **PA‐E3Y_h_** hydrogels appear to drive the formation of larger colonies than soft **PA‐E3Y_h_** hydrogels (Figure S4C, Supporting Information). Since PDAC epithelial tissues are organized into ducts, we assessed colony morphology to establish whether primary tumor histology is maintained. Indeed, after 14 days, the cultures acquired a duct‐like morphology in stiff **PA‐E3Y_h_** hydrogels (Figure 3H; Figure S4D, Supporting Information), resembling the ducts often found PDAC tissue (Figure 3I).

### 2.5 Stiffer Hydrogels Upregulate EMT and YAP Transcriptional Programs

**Figure 4:**
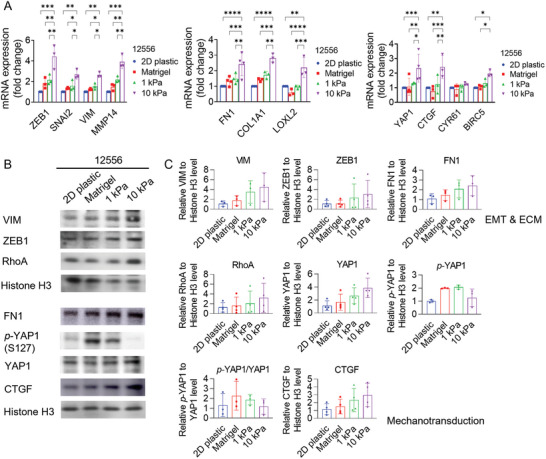
Matrix stiffness affects EMT phenotypes and mechanotransduction in PDAC. A) Relative expression of EMT, ECM related genes, as well as YAP1 and its target genes in PDAC cells (12556) cultured for 4 days on 2D plastic, in Matrigel, 1, and 10 kPa **PA‐E3Y**_**h**_ hydrogels, as measured by qPCR (*n* = 3). B,C) Western blot analysis of EMT, ECM and mechanotransduction related proteins expression in PDAC cells (12556) on 2D plastic, in Matrigel, 1, and 10 kPa **PA‐E3Y**_**h**_ hydrogels (*n* = 3). *P < 0.05; **P < 0.01; ***P < 0.001; ****P < 0.0001.

The EMT process plays a vital biological role in tumor invasion and metastasis. This process involves the loss of tight junctions and cell‐cell adhesion by malignant cells, thereby facilitating their migration beyond the primary tumor site.^[^ ^4^ ^]^ We investigated whether hydrogel stiffness regulates this process as well. PDAC cells encapsulated in stiff **PA‐E3Y_h_** hydrogels displayed higher expression of EMT markers (ZEB1, SNAI2, VIM, MMP14, LOXL2) at both mRNA (**Figure** **4**A; Figure S5A, Supporting Information) and protein levels (Figure 4B,C) compared to the softer hydrogel, Matrigel, and 2D culture. The elevated expression of MMP14 in stiff hydrogels is indicative of stiffness‐induced invasiveness, which promotes the migratory capability of the cancer cells to secondary sites. Similarly, LOXL2 is known to facilitate collagen crosslinking and has been correlated with desmoplasia, as well as increased resistance and metastasis in PDAC.^[^ ^38^ ^]^ These invasive and migratory behaviors are generally orchestrated by the activation of transcription factors *ZEB1* and *SNAI2*, which also induce upregulation of vimentin (VIM, Figure 4A–C; Figure S5A, Supporting Information). Additionally, we found that hydrogel stiffness had a significant impact on the expression of ECM genes, which are also markers of EMT. PDAC cells encapsulated in stiff **PA‐E3Y_h_** hydrogels displayed high expression of *COL1A1* and *FN1* at mRNA (Figure 4A; Figure S5A, Supporting Information) and protein level (Figure 4B,C). These molecules have been associated with poor prognosis^[^ ^39^ ^]^ and resistance to therapy.^[^ ^40^ ^]^ Hence, the data from our study suggests that matrix stiffness could potentially stimulate ECM remodeling associated with disease progression.

To ensure that the observed upregulated expression of EMT and ECM genes was not due to the higher concentration of Ca^2+^ ions in the stiff **PA‐E3Y_h_** hydrogels, we added the same amount of CaCl_2_ used to prepare stiff **PA‐E3Y_h_** hydrogels to PDAC cells cultured on a 2D plastic well‐plate under the same conditions (Figure S5B, Supporting Information). We found no evidence for increased EMT or ECM gene expression, suggesting that the elevated gene expression by PDAC cells in stiff **PA‐E3Y_h_** hydrogels indeed results from the interplay of the 3D framework, ECM‐like nanoscale architecture, and mechanical properties of stiff **PA‐E3Y_h_** hydrogels.

To explore how stiffness may control PDAC cell proliferation and transcriptional programs, we turned our attention to yes‐associated protein‐1 (YAP1), a prominent transcriptional coactivator in mechanotransduction,^[^ ^8^, ^41^ ^]^ which also plays a significant role in CSCs^[^ ^8^, ^41^ ^]^ by promoting their survival and self‐renewal.^[^ ^41^ ^]^ As expected, activation of YAP1 signaling was triggered by stiff substrates, leading to translocation of YAP1 from the cytoplasm (*p*‐YAP1) to the nucleus (nuclear YAP1). Total YAP1 levels positively correlated with increasing substrate stiffness while less *p*‐YAP1 was retained in the cytoplasm (Figure 4B,C). The lower expression of *p*‐YAP1 in stiff **PA‐E3Y_h_** hydrogels suggests that more YAP1 is translocated from the cytoplasm to the nucleus of PDAC cells encapsulated in the stiff hydrogels. Accordingly, the mRNA expression of *YAP1* and its transcriptional targets, *CTGF*, *CYR61*, and *BIRC5*, was found to be significantly upregulated in stiff **PA‐E3Y_h_** hydrogels (Figure 4A), compared to Matrigel and soft **PA‐E3Y_h_** hydrogels. At the protein level, both the upstream regulator of YAP1, RhoA, and its downstream target, CTGF, were upregulated in stiff **PA‐E3Y_h_** hydrogels (Figure 4B,C), suggesting that the canonical RhoA/YAP signaling axis is active in these conditions, which is associated with EMT and CSC phenotypes during tumor progression.^[^ ^42^ ^]^

### 2.6 Stiff **PA‐E3Y** **_h_** Hydrogels Enrich for Cancer Cells with More Aggressive Phenotypes

**Figure 5:**
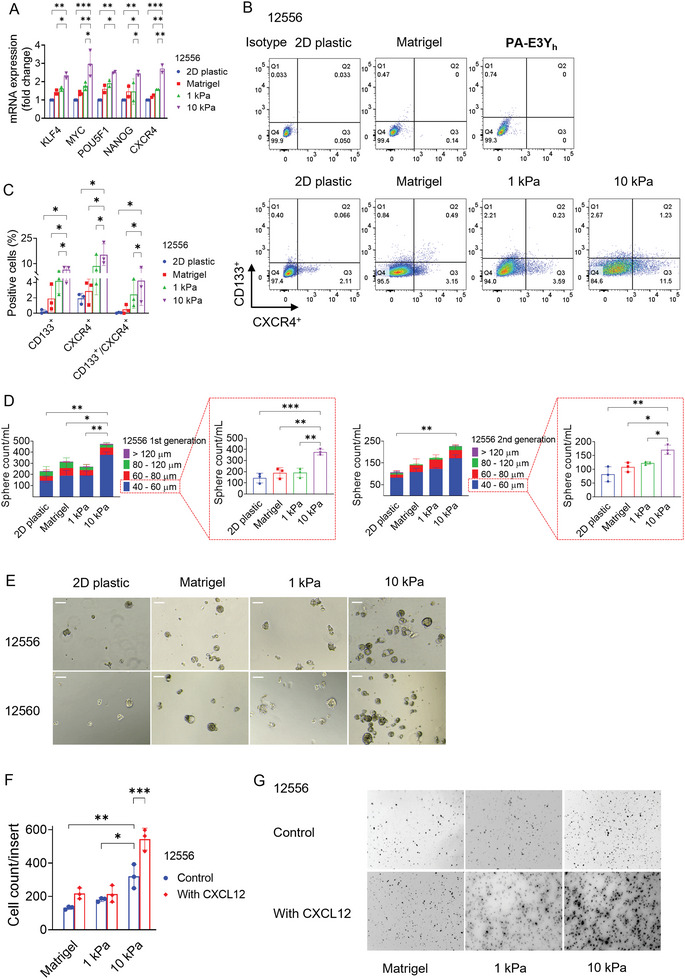
Matrix stiffness affects stemness phenotypes in PDAC. A) Relative expression of stemness‐related genes in PDAC cells (12556) cultured for 4 days on 2D plastic, in Matrigel, 1, and 10 kPa **PA‐E3Y**_**h**_ as measured by qPCR (*n* = 3). B,C) CD133^+^/CXCR4^+^ populations of PDAC cells (12556) cultured in 2D plastic, Matrigel, 1 kPa and 10 kPa **PA‐E3Y**_**h**_ hydrogels as measured by flow cytometry. Representative flow cytometry plots (B) are shown on the left, and the analysis (C) for the percentage of CD133^+^, CXCR4^+^, and CD133^+^/CXCR4^+^ populations is provided on the right side (*n* = 3). D) Number of first‐ and second‐generation spheres by size range formed by PDAC cells (12556) derived from 2D plastic, Matrigel, 1 and 10 kPa **PA‐E3Y**_**h**_ hydrogel (*n* = 3). E) Representative brightfield images of PDAC cell (12556 and 12560) spheres derived from 2D, Matrigel, 1 and 10 kPa **PA‐E3Y**_**h**_ hydrogels (scale bar: 100 µm). F) Relative invasion PDAC cells (12556) derived from 2D plastic, Matrigel, 1 and 10 kPa **PA‐E3Y**_**h**_ hydrogels as assayed in Matrigel‐coated transwell inserts, in the absence or presence of the chemoattractant CXCL12 (*n* = 3). G) Representative images of PDAC cells (12556) derived from 2D plastic, Matrigel and **PA‐E3Y**_**h**_ hydrogel, which invaded through Matrigel‐coated inserts with and without CXCL12. *P < 0.05; **P < 0.01; ***P < 0.001.

PDAC contains a subset of highly tumorigenic CSCs, which have been shown to drive tumor initiation, metastasis, and resistance to radio‐ and chemotherapy.^[^ ^23b^ ^]^ To investigate whether there is an enrichment of CSCs in stiff **PA‐E3Y_h_** hydrogels, we examined the expression of CSC‐related markers. As shown in **Figures** **5**A, S5A (Supporting Information), mRNA levels of KLF4, MYC, POU5F1, NANOG, and CXCR4 were upregulated 2 to 5‐fold in PDAC cells encapsulated in stiff **PA‐E3Y_h_** hydrogels compared to softer gels, Matrigel or 2D cultures. In addition, a higher proportion of CD133^+^, CXCR4^+^ and CD133^+^/CXCR4^+^ cell populations were detected by flow cytometry in stiff **PA‐E3Y_h_** hydrogels than in the other conditions (Figure 5B,C; Figure S5C, Supporting Information). CXCR4 is a marker of invasive cells, and its overexpression is associated with tumor aggressiveness and metastasis.^[^ ^43^ ^]^

To functionally validate the enrichment for CSCs and assess their self‐renewal ability, we studied spheroid formation in serum‐ and anchorage‐free culture conditions.^[^ ^44^ ^]^ As showed in Figure 5D,E, Figure S5D (Supporting Information), PDAC cells harvested from stiff **PA‐E3Y_h_** hydrogels formed more spheres, especially in the 40 to 60 µm diameter range, during their 1^st^ and 2^nd^ passage in serum‐ and anchorage‐free conditions compared to other conditions, providing functional proof for the enrichment of stem‐like cells.^[^ ^44^ ^]^

Next, we evaluated whether matrix stiffness also affects the invasive ability of PDAC cells, as predicted by the increase in EMT‐associated molecules (Figure 4; Figure S5A, Supporting Information), higher expression of CXCR4 and enrichment of a CXCR4^+^ PDAC cells (Figure 5A–C; Figure S5A,C, Supporting Information) in stiff **PA‐E3Y_h_** hydrogels. For this purpose, we extracted PDAC cells from **PA‐E3Y_h_** hydrogels and Matrigel followed by invasion assays in the presence or absence of the chemoattractant CXCL12, a specific ligand of the CXCR4 receptor. In the absence of a chemoattractant, approximately twice as many cells isolated from stiff **PA‐E3Y_h_** hydrogels migrated through the transwell compared to cells derived from soft hydrogels and Matrigel, respectively. This difference was further enhanced by a factor of three in the presence of the chemoattractant CXCL12 (Figure 5F,G; Figure S5E,F, Supporting Information). Therefore, PDAC cell cultures in stiff **PA‐E3Y_h_** hydrogels were enriched for genes and surface antigens associated with CSCs and displayed a higher invasive capacity, which is at least in part is mediated by the CXCR4/CXCL12 axis.

**Figure 6:**
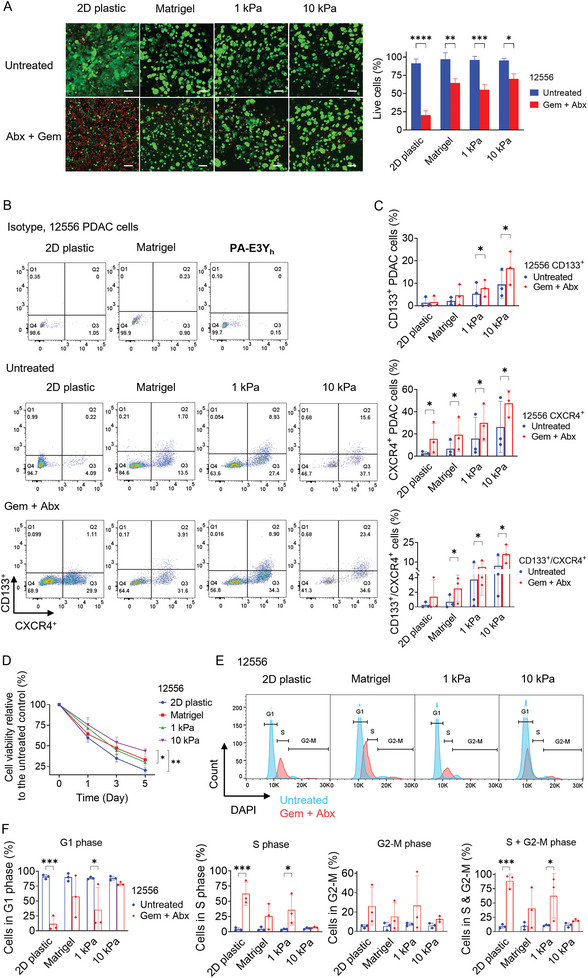
Matrix stiffness regulates stemness features and drug resistance. A) PDAC (12556) cell viability after gemcitabine (Gem) plus nab‐paclitaxel (Abx) treatment in different stiffness conditions. Live (green)/dead (red) cells assay was performed after 5 days of treatment with Gem/Abx in 2D, Matrigel, 1 and 10 kPa **PA‐E3Y**_**h**_ hydrogels (*n* = 3), scale bar: 100 µm. B,C) CD133^+^/CXCR4^+^ CSC population during drug treatment as evaluated by flow cytometry. B) Representative flow cytometry plots for CD133^+^, CXCR4^+^, and CD133^+^/CXCR4^+^ PDAC cells (12556) with and without drug treatment. C) Quantification of CD133^+^, CXCR4^+^ and CD133^+^/CXCR4^+^ CSC populations (*n* = 3). D) Normalized proliferation of PDAC cells (12556) from 2D plastic, Matrigel, 1 and 10 kPa **PA‐E3Y**_**h**_ hydrogels within 5 days Gem/Abx treatment (*n* = 3). E,F) Response of PDAC cell cycle to Gem/Abx in 2D, Matrigel, 1 and 10 kPa **PA‐E3Y**_**h**_ hydrogels. E) Representative cell cycle profiles of untreated (blue) and treated (red) PDAC cells (12556) gated to exclude debris and doublets. F) Percentages of PDAC cells (12556) that were in G1, S, G2‐M and S+G2‐M phases compared to untreated controls (*n* = 3). *P < 0.05; **P < 0.01; ***P < 0.001; ****P < 0.0001.

Since CSCs are inherently resistant to chemotherapy, we explored whether hydrogel stiffness impacts the chemosensitivity of PDAC cells. We tested their response to a 5‐days course of the current standard‐of‐care treatment for PDAC: Gemcitabine (a nucleoside analog) and Abraxane (nab‐paclitaxel, a microtubule stabilizer).^[^ ^45^ ^]^ Live/dead staining confirmed that PDAC cells cultured in stiff **PA‐E3Y_h_** hydrogels showed a higher percentage of cell viability (70%) following drug treatment (**Figure** **6**A), compared to 64% and 55% in Matrigel and soft **PA‐E3Y_h_** hydrogels, respectively. On 2D plastic, viability was dramatically reduced to 20%. While drug delivery could be slightly reduced in 3D hydrogels and Matrigel compared to 2D plastic,^[^ ^12^ ^]^ the enhanced cell viability of cells in stiff **PA‐E3Y_h_** hydrogels over soft **PA‐E3Y_h_** hydrogels is more likely to result from cell‐intrinsic changes in chemoresistance, e.g. stiffness‐induced enrichment for CSCs (Figure 6B,C; Figure S6A,B, Supporting Information). To corroborate this, we performed flow cytometry analysis for the CSC markers CD133 and CXCR4. As expected, for all conditions (2D plastic, Matrigel, soft and stiff **PA‐E3Y_h_** hydrogels) CD133^+^/CXCR4^+^ positive cells were enriched after treatment with Abraxane‐Gemcitabine for 5 days due to their inherent chemoresistance (Figure 6B,C; Figure S6A,B, Supporting Information). The highest fraction of CD133^+^/CXCR4^+^ positive cells was found in stiff **PA‐E3Y_h_** hydrogels, suggesting that stiffness‐induced enrichment for CSCs is a key contributor to chemotherapeutic resistance in PDAC. We also measured cell viability over a week by resazurin reduction, which we normalized to untreated controls; the resulting data confirm that stiff hydrogels promote PDAC cell survival (Figure 6D).

In order to evaluate treatment response mechanistically, the cultures were analyzed by cell cycle profiling (Figure 6E,F; Figure S6C,D, Supporting Information). Gemcitabine and Abraxane treatment results in most cells resting in S and G2/M phase.^[^ ^19^ ^]^ Thus, the percentage of cells in S and G2/M phase following drug treatment should reflect drug sensitivity of PDAC cells when cultured in different stiffness conditions. Compared to PDAC cells cultured in 2D plastic, Matrigel and 1 kPa hydrogels, 10 kPa **PA‐E3Y_h_** hydrogel showed the lowest proportion of cells residing in S and G2/M phases, and the highest percentage of cells remaining in G1 phase (Figure 6E,F; Figure S6C,D, Supporting Information). These findings indicate that PDAC cells cultured in stiff **PA‐E3Y_h_** hydrogels become inherently resistance to chemotherapy‐induced cell cycle arrest.

### 2.7 Discussion

We have developed a biocompatible and tunable 3D self‐assembling hydrogel to determine the impact of matrix stiffness on malignant cell phenotypes and drug resistance, using highly desmoplastic and invasive PDAC as our model system. These hydrogels displayed reproducible and tunable stiffness, which was enhanced using a heating‐cooling cycling protocol. This method effectively modulated the properties of the colloidal phase of **PA‐E3Y_,_** including viscosity and molecular conformation, as previously described with different gels.^[^ ^32b^ ^]^ Using a fixed gelator concentration against various concentrations of aqueous solutions of CaCl_2,_ the obtained solutions of **PA‐E3Y (PA‐E3Y_h_**) formed hydrogels with stiffness ranging several orders of magnitude and thereby spanning the stiffness observed for healthy pancreas as well as PDAC tissues. Moreover, **PA‐E3Y_h_** hydrogels displayed self‐recovery, which makes them suitable for biomedical applications requiring self‐recovery after deformation such as 3D cell cultures for mechanobiology assays or injectable carriers and therapeutics for in vivo studies.^[^ ^46^ ^]^

Previous studies have shown that the mechanical properties of PA gels can be tuned through several strategies including modification of β‐sheet‐forming regions,^[^ ^47^ ^]^ incorporation of photo‐crosslinkable segments^[^ ^48^ ^]^ or host‐guest moieties.^[^ ^49^ ^]^ However, none of these strategies can create hydrogels with a broad range of stiffness without using toxic crosslinking agents such as glutaraldehyde or laborious multi‐step chemical synthesis. In contrast, **PA‐E3Y_h_** provides a straightforward approach to modulate hydrogel stiffness with the simple addition of CaCl_2_. The mechanical tuneability of the hydrogels likely results from the synergistic interactions of multiple non‐covalent interactions including hydrogen bonds, hydrophobic interactions, metal coordination, and π‐π interactions, as well as conformational changes that drive **PA‐E3Y_h_** self‐assembly. As previously shown for similar self‐assembling matrices,^[^ ^19^, ^20^ ^]^ **PA‐E3Y_h_** self‐assembled into a network of fibers that resemble the architecture of native PDAC tumor ECM. The microstructure of the ECM is known to facilitate cell‐matrix interactions, cell‐cell communication, and mechanotransduction.^[^ ^50^ ^]^ As such, these new ECM‐mimicking hydrogels constitute overcoming the poor mechanical properties, narrow stiffness range, molecular complexity, high costs, and batch‐to‐batch variation of commonly used biomaterials for 3D cell culture and disease modeling such as Matrigel.

Our results reveal that stiffening of **PA‐E3Y_h_** hydrogels to physiologically relevant in vivo levels maintain PDAC cell viability in the same range as other 3D platforms while enhancing the ability of the cells to survive in suspension and grow into tumor spheres. The self‐assembled cultures formed luminal structures comparable to those of Matrigel‐embedded organoids, indicating that the ductal nature of the primary PDAC cells is preserved as well. The acquisition of stem‐like and mesenchymal features by these cultures correlated with hydrogel stiffness, which broadly agrees with the literature.^[^ ^51^ ^]^ Mechanistically, **PA‐E3Y_h_** stiffening appears to enhance aggressiveness via YAP1 nuclear translocation, to activate RhoA/YAP1 signaling and to increase expression of downstream targets such as CTGF. Functionally, stiffening to levels found in vivo leads to a substantial enrichment in CD133^+^/CXCR4^+^ CSCs, which display increased invasive ability upon CXCL12‐mediated stimulation. This PDAC cell subpopulation is known to be among the most resistant to chemotherapy in PDAC^[^ ^43^ ^]^ and our results show that they are enriched upon treatment in stiffer hydrogels, which translates into higher cell survival compared to softer hydrogels. The observed chemoresistance appears to be intrinsic rather than extrinsic as demonstrated by cell cycle profiling. It remains to be seen whether stiffness in these hydrogels also promotes resistance to drugs with alternative mechanisms of action.

These findings highlight how tailoring stiffness to that of the in vivo TME results in more realistic treatment responses. Furthermore, the tuneability of stiffness may also enable recreation of the TME at different stages of cancer progression. A next step to validate this concept may be to assess whether patient‐specific stiffness platforms mimic in vivo cancer cell drug response better than existing non‐tunable platforms such as Matrigel, with a larger cohort and a wider spectrum of drugs. One of the advantages of models with tunable self‐assembly such as **PA‐E3Y_h_** is that they enable the systematic study of multiple variables simultaneously and study their interplay. For example, in future studies one may investigate whether substrate stiffening correlates with other extrinsic determinants of response such as immune infiltration and vascularization. Along these lines, **PA‐E3Y_h_** hydrogels may be a particularly useful system to study the contradictory effects of the tumor microenvironment (TME) on PDAC. Such tumor‐promoting and tumor‐restraining effects have led to the failure of numerous stroma‐directed therapies.^[^ ^52^ ^]^ Here, we show that PDAC cells preferentially migrate when cultured on stiffer substrates. In vivo, the stiff matrix often forms dense tracts of interstitial collagen (“collagen highways”) along which cell migration has been observed. PA hydrogels with tunable stiffness enable us to test which subpopulations of cells are more likely to invade surrounding tissue along these fibers and whether they do so collectively or as single cells.^[^ ^53^ ^]^ CD133^+^/CXCR4^+^ CSCs are a prime candidate since the CXCR4 ligand, CXCL12, has been shown to be a critical homing factor in the premetastatic niche.^[^ ^54^ ^]^ Implanting these models into animals paves the way for in vivo investigations into how stiffness impacts tumor growth, potentially corroborating our results. In this context, we have previously demonstrated that PDAC cells exhibit a greater tumor engraftment rate in mice when xenografted with 1 kPa PA hydrogels compared to 100 Pa Matrigel.^[^ ^19^ ^]^ The use of **PA‐E3Y_h_** will enable us to examine the scaling of this effect across various degrees of magnitude.

In acknowledging the potential limitations inherent in our methodology, we recognize the multifaceted role of Ca^2+^ ions in various cellular processes, necessitating extensive future research for a comprehensive understanding of these interactions. Ca^2+^ ions are integral in a spectrum of physiological and pathological states, significantly influencing cellular dynamics such as migration, which is pertinent to the aggressiveness of PDAC.^[^ ^55^ ^]^ Consequently, the potential impact of Ca^2+^ signaling on tumor biology must be carefully considered in the interpretation of our findings. Furthermore, there exists a discrepancy between the degradation processes of PA fibers and the native ECM by cancer cells. Given the pivotal role of matrix remodeling in tumor progression and metastasis, this represents a potential limitation of our approach. Additionally, it is imperative to acknowledge that the **PA‐E3Y_h_** hydrogels utilized in our study may not entirely replicate the intricate complexities of the in vivo ECM, particularly its microarchitecture and the release of matrikines. Thus, the mechanisms driving ECM alterations in our model may diverge to some extent from those in the natural in vivo environment of PDAC patients. Finally, to gain a comprehensive insight into the molecular mechanisms that contribute to enhanced chemoresistance induced by stiffer gels, it will be imperative to conduct detailed investigations to ascertain the degree to which YAP1 and/or ROCK influence the observed phenotypes and responses.

## 3 Conclusion

Altogether, tunable **PA‐E3Y_h_** hydrogels constitute a suitable in vitro platform for modeling stiffness‐mediated invasion and stemness‐related phenotypes. The possibility of customizing the stiffness of the hydrogels over a wide physiological range combined with their intrinsic ECM‐like fibrous architecture presents an opportunity to investigate the effects of matrix stiffness on disease development. Key ECM components such as collagen, fibronectin, and hyaluronan, which contribute to tissue stiffening, have been shown to promote niche‐dependent phenotypes, such as stemness and EMT in PDAC.^[^ ^19^ ^]^ Co‐assembly of **PA‐E3Y_h_** with these molecules may allow the study of their role in mechanotransduction in CSC‐enriched PDAC cultures. Likewise, the inclusion of additional cell types such as fibroblasts and macrophages ought to further recapitulate the TME in the hydrogels. This is especially relevant since stromal cells deposit the majority of the interstitial ECM, secrete CXCL12 among other regulatory cytokines^[^ ^56^ ^]^ and actively compress cancer cells as well.^[^ ^14^ ^]^ Their incorporation into the **PA‐E3Y_h_** model may further help determine the discrete and collaborative roles of tissue mechanics and tissue composition during tumor initiation and progression. Additionally, the wider range of hydrogel stiffness offered by **PA‐E3Y_h_** opens up potential avenues of research in mechanobiology, such as examining the influence of matrix stiffness on stem cell expansion and organoid formation,^[^ ^57^ ^]^ as well as its impact on adipogenesis in MSCs.^[^ ^58^ ^]^

## 4 Experimental Section

### PDX‐Derived PDAC Cells

Primary pancreatic cancer tissue was collected by the CAM‐PaC (Integrative Analysis of Gene Functions in Cellular and Animal Models of Pancreatic Cancer) consortium under grant agreement no. 602 783. Tissue samples were obtained from the ARC‐Net biobank of the University and Hospital Trust of Verona under Program 1885 protocol 52 438 23/11/2010 and project approval program 2172 protocol 26 773 23/05/2012, approved by the Verona University Hospital Ethics Committee. Patient‐derived tumor xenografts (PDX) were produced under the ministerial decree no. 107/2012‐B and 108/2012‐B issued by the Ministry of Health based on the legislative decree 106/92 regarding the protection of animals used in scientific research.

Isolation of primary human PDAC cells from the PDXs for the in vitro studies was performed as previously described.^[^ ^59^ ^]^ Briefly tissues were minced into small pieces which were subsequently digested using a mix of collagenase P (4 mg mL^−1^, Sigma) and Dispase II (1 mg mL^−1^, PluriSTEM Dispase, Millipore) in medium containing 2.5% FBS, Penicillin/Streptomycin, NEAAs, L‐Glutamine and Na‐pyruvate for 1–2 hours at 37 °C under rotation/shaking. Following dissociation, the cells (12556, 12560) were washed and filtered using a 40 µm mesh three times and were counted and seeded in adherent or 3D conditions. PDAC cells were expanded in RPMI medium (Life Technologies, UK) supplemented with 10% fetal bovine serum at 37 °C and 5% CO_2_. For the experiments, cells were maintained in serum‐free sphere medium: DMEM/F12 supplemented with 2% B27 (Life Technologies, UK), 20 ng mL^−1^ FGF2 (PeproTech, UK) and 2 mM L‐glutamine (Life Technologies, UK).

### Cell Encapsulation in **PA‐E3Y** **_h_** Hydrogel

To create hydrogels of **PA‐E3Y**, first we prepared an aqueous solution of the gelator at 1 mg 100 µL^−1^ (**1% wt, PA‐E3Y**). Negatively charged **1% PA‐E3Y** (1 mg 100 µL^−1^, 1% wt) was dissolved in HEPES buffer (10 mM HEPES, 3 mM KCl, 150 mM NaCl), adjust pH to 7.2 – 7.4 with 0.1 M NaOH. In light of previous reports where pre‐heating and cooling of a gelator solution significantly enhanced the stiffness of self‐assembling hydrogels created with Ca^2+^ ions, we first heated an aqueous solution of **PA‐E3Y** to 80 °C followed by cooling to room temperature (**PA‐E3Y_h_**). **PA‐E3Y_h_** solutions were mixed with cells (4 000 cells µL^−1^). **PA‐E3Y_h_**‐cell mixture were added into CaCl_2_ solution according to the volume ratio of PA and CaCl_2_ at 3:1. **PA‐E3Y_h_** were self‐assembled in CaCl_2_ at 5 mM and 50 mM (the final concentration of Ca^2+^ is 1.25 mM and 12.5 mM), corresponding to 1 and 10 kPa, respectively. The cell laden **PA‐E3Y_h_** hydrogel was incubated at 37 °C to form a homogeneous hydrogel. For experiments, the sphere medium was added into the well‐plate, and cells were cultured at 37 °C in a humidified atmosphere of 5% CO_2_.

### Drug Treatments

PDAC cells cultured in 2D, Matrigel and **PA‐E3Y_h_** hydrogels for 48 h were treated with combination of Gemcitabine (100 ng mL^−1^) and Abraxane (10 µM) for 5 days. These concentrations were demonstrated previously to reveal variations in chemoresistance among 3D culture models of PDAC.^[^ ^19^ ^]^

### Atomic Force Microscopy

Frozen PDX tissue blocks were cut into 20‐µm‐thick sections. Before Atomic force microscopy (AFM) measurement, the cryosection was immersed in PBS and thawed at room temperature. The stiffness of PDX measurements were conducted on a JPK Nanowizard‐4 (JPK Instruments, Germany) mounted on an inverted optical microscope (IX‐81; Olympus, Japan). AFM pyramidal cantilever (MLCT; Bruker, MA, USA) with a 20 nm radius pyramidal tip, a front angle of 15 ± 2.5°, and a spring constant of 0.07 N m^−1^ was chosen for the ability to address Young's modulus between 0.1 and 100 kPa. Force‐curves were acquired in the force spectroscopy mode with a setpoint force of 2 nN, a speed of 2 µm s^−1^, and a Z length of 10 µm. The Young's modulus was extracted over a depth of 500 nm, ensuring sufficient indentation of the actin network and cytoplasm.^[^ ^60^ ^]^ Six PDXs and four MDAs specimens were used for stiffness measurement, and AFM was performed on three separate cryosections for each specimen. Five AFM force maps were obtained on each cryosection, and every force map covered a size of 50 × 50 µm^2^ region under 10 × 10 point grids representing 100 force curves. Young's modulus (E) was calculated from the force‐distance curves by fitting the contact region of the approach curve with the Hertz contact model using the AFM software (JPK).

### Proliferation Assay

Cell proliferation in **PA‐E3Y_h_** hydrogel was measured by resazurin reduction assay every 2 days. Resazurin (alamarBlue Cell Viability Reagent, DAL1025, Invitrogen, US) was diluted 1:10 in PBS and added into the multi‐well plates in a ratio of 1:1 with cell culture medium, and the culture was incubated at 37 °C for 5 h. Following the incubation, the supernatant solution was collected and transferred into a suitable optic multi‐well plate, and the resulting fluorescence was measured on a FLUOstar OPTIMA microplate reader (BMG Labtech, Germany) with filters for excitation at 544 nm and emission at 590 nm for quantification. The results were further normalized by subtracting the value of acellular controls.

### Spheroid Formation Assay

Spheroid formation assay was performed as previously detailed.^[^ ^61^ ^]^ PDAC cells were dissociated into single cell from different stiffness conditions by Trypsin (for 2D culture, Sigma‐Aldrich, US) and TrypLE Express (for Matrigel and **PA‐E3Y_h_** hydrogels, Gibco, USA), respectively. PDAC spheres were generated in DMEM‐F12 containing B‐27 (GIBCO) and bFGF (Peprotech) in low attachment 24‐well plates (Corning, US) after 7 days following initial seeding of 2 000 PDAC cells. For serial passaging to secondary generations spheres were collected through a 40 µm strainer (Sysmex, Germany) and following trypsinization were seeded again in the same conditions. On day 7 and day 14, the number of spheres in the different diameter size fractions was quantified using a CASY cell counter (Roche Applied Sciences, Germany). Spheres were imaged using an Olympus CKX41 bright‐field microscope equipped with an Infinity 3 camera (Lumenera, US).

### Invasion Assay

Invasion of cells in 3D was assayed in 24 well 6.5 mm transwell with 8 µm polycarbonate membrane inserts (Corning, US), which were coated with 50 µL of Matrigel (Corning, US). PDAC cells were extracted after culture in different stiffness conditions, and 5 × 10^4^ cells with 400 µL of serum‐free medium were seeded into the transwell insert. After starving the cells for 12 h in serum‐free medium in both the upper insert and the bottom chamber, the medium in the bottom chamber was replaced with 700 µL of 10% FBS‐containing medium, creating a serum gradient to attract cells. In the CXCL12 group, the medium in the bottom chamber was replaced with 10% FBS‐containing medium supplemented with 100 ng mL^−1^ CXCL12 (PeproTech EC). Assay chambers were incubated for 24 h at 37 °C in a humidified atmosphere of 5% CO_2_. Invaded cells were fixed with 4% paraformaldehyde (PFA, g mL^−1^), and the Matrigel coating was removed by wiping with cotton buds. The invaded cells on the underside of the insert that transmigrated through the Matrigel layer, were stained with 10 µg mL^−1^ DAPI and imaged on an Olympus CKX41 microscope equipped with a CKX‐RFA fluorescence illuminator (Olympus, Japan) and an Inifinity 3 camera (Lumenera, US). Total cell numbers were analyzed on ImageJ (NIH, US).

### Flow Cytometry

PDAC cells were extracted by dissociating **PA‐E3Y_h_** hydrogels and Matrigel in TrypLE Express (Gibco, USA) for 20 min incubation. PDAC cells (10^6^ cells mL^−1^) were blocked with Flebogamma (Grifols, Spain) for 45 min in 96‐well plates with V‐shaped bottoms and incubated with fluorescently labelled antibodies for 1 hour at 4 °C in the dark. DAPI was used to exclude dead cells and the data were collected on an LSRFortessa cell analyzer (BD Biosciences, USA). Antibodies and their respective dilutions were listed here: anti‐CD133/1 (AC133)‐PE (human 1:100, 130‐080‐801, Miltenyi Biotec); anti‐CXCR4‐APC (human, 1:100, 306 510, BioLegend), and their corresponding isotype controls. All reactions and manipulations were performed in sorting buffer (3% BSA in 1× PBS). For cell cycle analysis, cells were fixed with 4% paraformaldehyde, permeabilized with 0.25% Triton X‐100 and stained with DAPI (10 µg mL^−1^). Cells were analyzed on an LSRFortessa cell analyzer (BD Biosciences, USA) and gated to exclude debris and doublets (Figure S7, Supporting Information).

### Western Blot

Cell lysates were prepared in RIPA buffer and protein separation was achieved in pre‐cast 4–12% Bis‐Tris NuPAGE gels (1.0 mm, Invitrogen, UK) with 1× MOPS running buffer. Following blotting onto a PVDF membrane, the membranes were blocked in 5% BSA for 1 h and incubated with primary antibodies overnight at 4 °C. Detection of the signal was performed on the second day following incubation with HRP‐conjugated antibodies and development with ECL Prime western blotting detection reagent (GE Healthcare, US). The resulting chemiluminescent signal and image was detected on an Amersham Imager 600 (GE Healthcare, US). The primary antibodies and their respective dilutions were: YAP1 (SC‐101199, 1:200, Santa Cruz); *p*‐YAP1 (S127, 1:10 000, ab76252, Abcam); CTGF (1:1000, ab6992, Abcam); RhoA (1:1000, ab54835, Abcam); Vimentin (1:1000, ab92547, Abcam); Histone H3 [EPR16987] (1:400, ab176842, Abcam).

### Solid‐Phase Peptide Synthesis

PAs were synthesized by solid‐phase peptide synthesis from a Rink amide 4‐methylbenzhydrylamine polymer‐bound resin (Sigma, US) on a Liberty Blue instrument (CEM, UK). To synthesize 1 mmol of peptide, 4 mmol of Fmoc‐protected amino acids (Sigma, US) were coupled by 4 mmol 1‐hydroxybenzotriazole (HOBt) and 6 mmol *N,N*′‐diisopropyl‐carbodiimide (DIC) in N,N‐dimethylformamide (DMF). Amino acids were deprotected in 20% piperidine in DMF. The N‐terminus of the peptide was coupled to a palmitoyl tail by adding 4 mmol of palmitic acid with 4 mmol HOBt and 6 mmol DIC in a 1:1 solution of DMF and dichloromethane. Complete coupling of the tail was verified by ninhydrin reaction (Kaiser test). Final cleavage of the resin was achieved with a solution of 95% trifluoracetic acid (TFA), 2.5% triisopropylsilane and 2.5% water for 3 h at room temperature. Residual TFA was removed by rotary evaporation and peptides were precipitated in cold diethyl ether. Crude products were then freeze‐dried overnight. PAs were then purified by reverse‐phase HPLC (Figure S1A, Supporting Information) and verified by mass spectrometry (Figure S1B, Supporting Information).

### Mechanical Characterization of PA Gel and Matrigel

The mechanical properties of PA hydrogel and Matrigel were measured with a Discovery HR‐3 rheometer (TA Instruments, USA). 45 µL of PA gel or Matrigel were placed on the center of the bottom plate, and the upper geometry with 8 mm diameter was lowered to a gap of 250 µm. Measurements were performed by amplitude sweep and frequency sweep at 25 °C, and a constant frequency of 1 Hz in the 0.01 – 10% strain during the amplitude sweep, while the oscillation frequency experiments were carried out at a 0.1% fixed strain along 0.1–100 Hz. The shear modulus (G) was obtained from the rheometer, and calculated by the equation

(1) $$G=\sqrt{{G^{\prime }}^{2}+{G^{\prime \prime }}^{2}}$$

and then converted to Young's modulus (E) by the equation

(2) $$E=\;2G\left(1+\upsilon \right)$$

where G′ is the shear storage modulus, G″ is the shear loss modulus, and υ is Poisson's ratio of material, ≈0.5.

The self‐recovery of **PA‐E3Y_h_** hydrogels was monitored as follows: an initial 0.1% strain was held for the first 100 s, then it was increased to 100% for 100 s, followed by a recovery segment of 0.1% stress for 200 s, increased to 100% for 200 s, and then continuous shear force of 0.1% strain for 400 s. Self‐recovery was calculated by the ratio of G’ in the third cycle to G’ in the initial cycle.

### Circular Dichroism Spectroscopy

Circular dichroism (CD) was measured with a Chirascan circular dichroism spectrometer (Applied Photophysic Limited, UK). A solution of **PA‐E3Y_h_** (0.01% wt) with CaCl_2_ (0.05 mM or 0.5 mM) was added into a quartz cuvette with a 1 mm path length. The parameters were set up as follows: 0.5 nm data pitch; continuous scanning mode; scanning speed at 100 nm min^−1^; 2 nm bandwidth; five times accumulation. CD spectra were recorded by signal integrating three scans, monitored at a 2 min interval from 190 to 260 nm at a speed of 50 nm min^−1^. Blanks (HEPES buffer only) were run to subtract noise from readings. Further noise reduction was achieved by applying a smoothing factor of 10. All CD data are presented as ellipticity and recorded in millidegree (mdeg), and spectroscopic data were analyzed on Pro‐Data Viewer (Applied Photophysics, UK).

### Thioflavin T Fluorescence Assay

Thioflavin T (ThT) fluorescence assay was used to analyze β‐sheet formation. A solution of **PA‐E3Y_h_** (0.2% wt) with CaCl_2_ (1 mM or 10 mM) was mixed with 20 µL of ThT (0.4 mM). Each sample was mixed by pipetting up and down three times, loaded into a 96‐well plate, sealed with paraffin film and incubated for 1 h. The 96‐well plates were kept in humidified Petri dishes to prevent evaporation. Self‐assembled nanofibers were imaged on a confocal microscope using excitation and emission wavelengths of 458 and 468 nm, respectively. **PA‐E3Y_h_** and CaCl_2_ solutions without ThT were used as control and did not exhibit any fluorescent signal.

### Scanning Electron Microscopy

**PA‐E3Y_h_** hydrogels (1 mg 100 µL^−1^, 1% wt) assembled with CaCl_2_ (5 mM or 50 mM) without cells and decellularized pancreatic cancer PDX tissue were imaged by scanning electron microscopy (SEM). Samples were fixed for 3 h with 2.5% glutaraldehyde in water at room temperature. The hydrogels were gradually dehydrated with increasing concentrations of ethanol (20, 50, 70, 80, 90, 96, and 100%, v/v), twice per solution for 10 min. Dehydrated samples were then subjected to critical point drying (K850, Quorum Technologies, UK). SEM micrographs of the xerogels were acquired on Inspect F50 (FEI Comp, the Netherlands) after sputter‐coating with gold (10 nm thick).

Energy‐dispersive X‐ray spectroscopy (EDX) was a classic method used in combination with SEM and enables to analyze the near‐surface elements at trace amount, such as the elemental composition of each point and amount of the interested position from the imaged area. Combined with SEM, a voltage of 10–20 keV was applied to produce energy of beam to cause X‐ray emission, and the presence of the atomic composition, such as Ca, in the fiber or flake of the hydrogel were detected.

### Statistical Analysis

Data are shown as mean ± standard deviation, unless otherwise specified. Multiple comparisons were performed by one‐way or two‐way ANOVA with Bonferroni correction in Prism 9.0 (GraphPad, USA). The multiple comparisons in Figures 2C,4H and Figures S2D,S5D (Supporting Information) were performed by one‐way ANOVA, the rest were analyzed by two‐way ANOVA. Two groups comparisons were performed by an unpaired and paired t‐test. The two groups comparisons in Figure 1B,5C, Figure 6B were performed by paired t‐test, the rest were analyzed by unpaired t‐test. The significance threshold was set at *P* < 0.05. All experiments were performed three independent times with two or three technical replicates.

### Ethics Approval Statement

Tissue and primary cell work presented here was done with ethical approval, protocols, and agreements in place, as detailed in Experimental Section.

### Patient Consent Statement

Informed consent was obtained from all participants through the ARC‐Net Biobank of the University and Hospital Trust of Verona approved by the Verona University Hospital Ethics Committee.

## Conflict of Interest

The authors declare no conflict of interest.

## Author Contributions

Conceptualization was done by C.H., A.M., O.M.T.P., and B.O.O.. Methodology was applied by Y.L., B.O.O., D.O.P., W.Q.L., M.L.L., S.T., W.W., M.T., M.K., D.L., R.T.L., and A.S.. Investigation was carried out by Y.L. and B.O.O.. Y.L., B.O.O., D.O.P., and W.Q.L. dealt with formal analysis. Visualization was conducted by Y.L.. Supervision was done by O.M.T.P., C.H., and A.M.. Y.L. and B.O.O. dealt with Writing—original draft. Writing—review & editing was done by O.M.T.P., C.H., A.M., B.O.O., D.L., M.T., D.O.P., W.Q.L., and M.L.L..

## Supporting information

Supporting Information

## Data Availability

The data that support the findings of this study are available from the corresponding author upon reasonable request.
