# Supplementary material for: A Self‐Assembled 3D Model Demonstrates How Stiffness Educates Tumor Cell Phenotypes and Therapy Resistance in Pancreatic Cancer
Source: Adv Healthc Mater. 2024 Mar 22;13(17):2301941. doi: 10.1002/adhm.202301941 (PMC11468796; doi:10.1002/adhm.202301941)
Supplement: Supplementary file 1 — Supporting Information [file ADHM-13-2301941-s001.pdf]

ADVANCED HEALTHCARE MATERIALS

Supporting Information

for *Adv. Healthcare Mater.*, DOI 10.1002/adhm.202301941

A Self-Assembled 3D Model Demonstrates How Stiffness Educates Tumor Cell Phenotypes and Therapy Resistance in Pancreatic Cancer

Ying Liu, Babatunde O. Okesola, David Osuna de la Peña, Weiqi Li, Meng-Lay Lin, Sara Trabulo, Marianthi Tatari, Rita T. Lawlor, Aldo Scarpa, Wen Wang, Martin Knight, Daniela Loessner, Christopher Heeschen, Alvaro Mata* and Oliver M. T. Pearce**

Supporting Information

A self-assembled 3D model demonstrates how stiffness educates tumor cell phenotypes and therapy resistance in pancreatic cancer

Ying Liu[1,2], Babatunde O. Okesola[3], David Osuna de la Peña[1,2], Weiqi Li[2], Meng-Lay Lin[1], Sara Maria David Trabulo[1], Marianthi Tatari[1], Rita T. Lawlor[4,5], Aldo Scarpa[4,5], Wen Wang[2], Martin Knight[6,7], Daniela Loessner[1,8,9,10], Christopher Heeschen[11,12], Alvaro Mata[13,14,15]*, Oliver M. T. Pearce[1]**

This PDF file includes:

Figs. S1 to S8

A

B

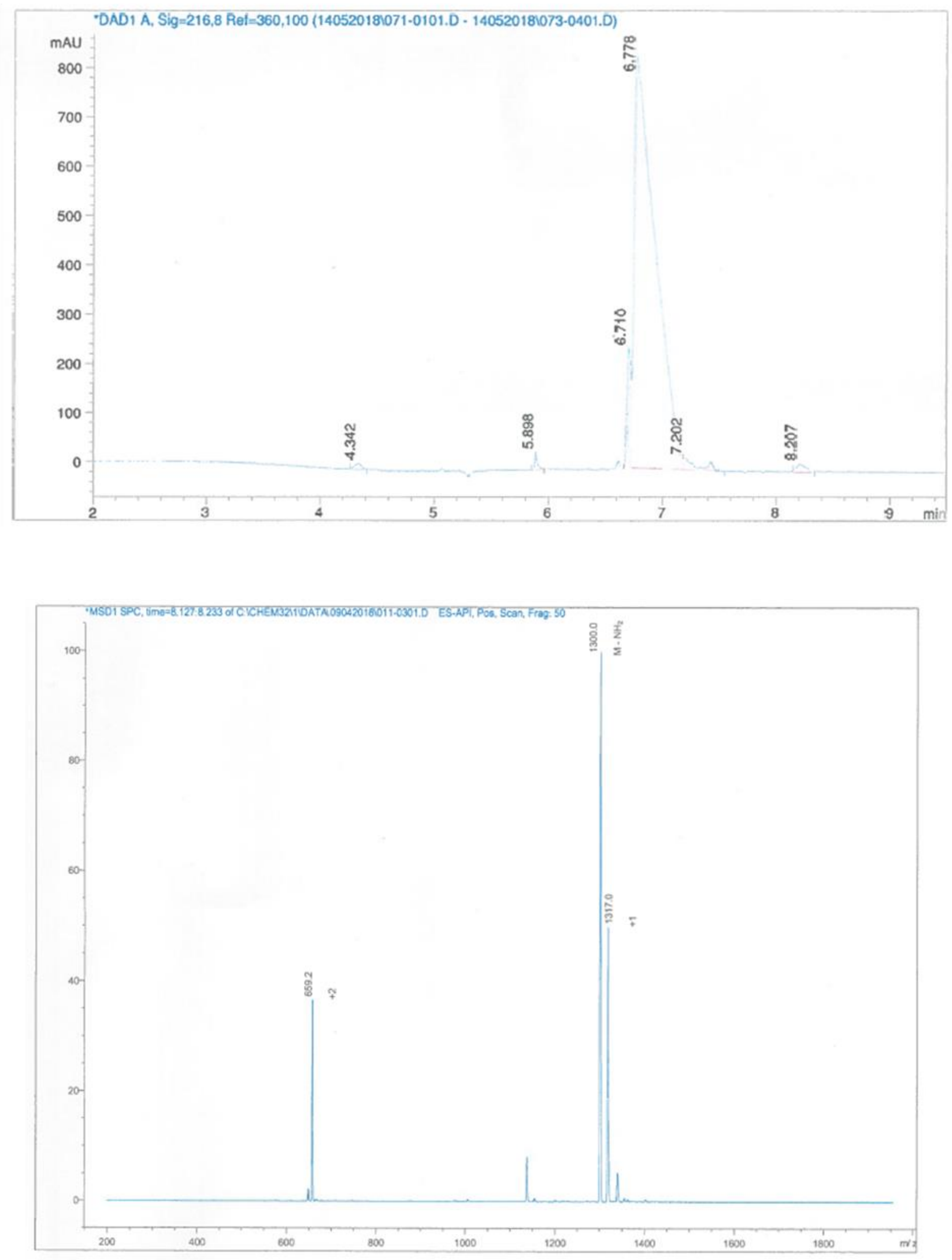

Figure S1. Molecular characterization of **PA-E3Y**. (**A**) Analytical reverse-phase high-performance liquid chromatography (RP-HPLC) trace of **PA-E3Y** (C_{16}-V3A3E3Y) at 220 nm. Gradient: Acetonitrile–Water (+0.1% $NH4^{+}$). (**B**) Electrospray ionization mass spectrum (ESI-MS) of **PA-E3Y**.

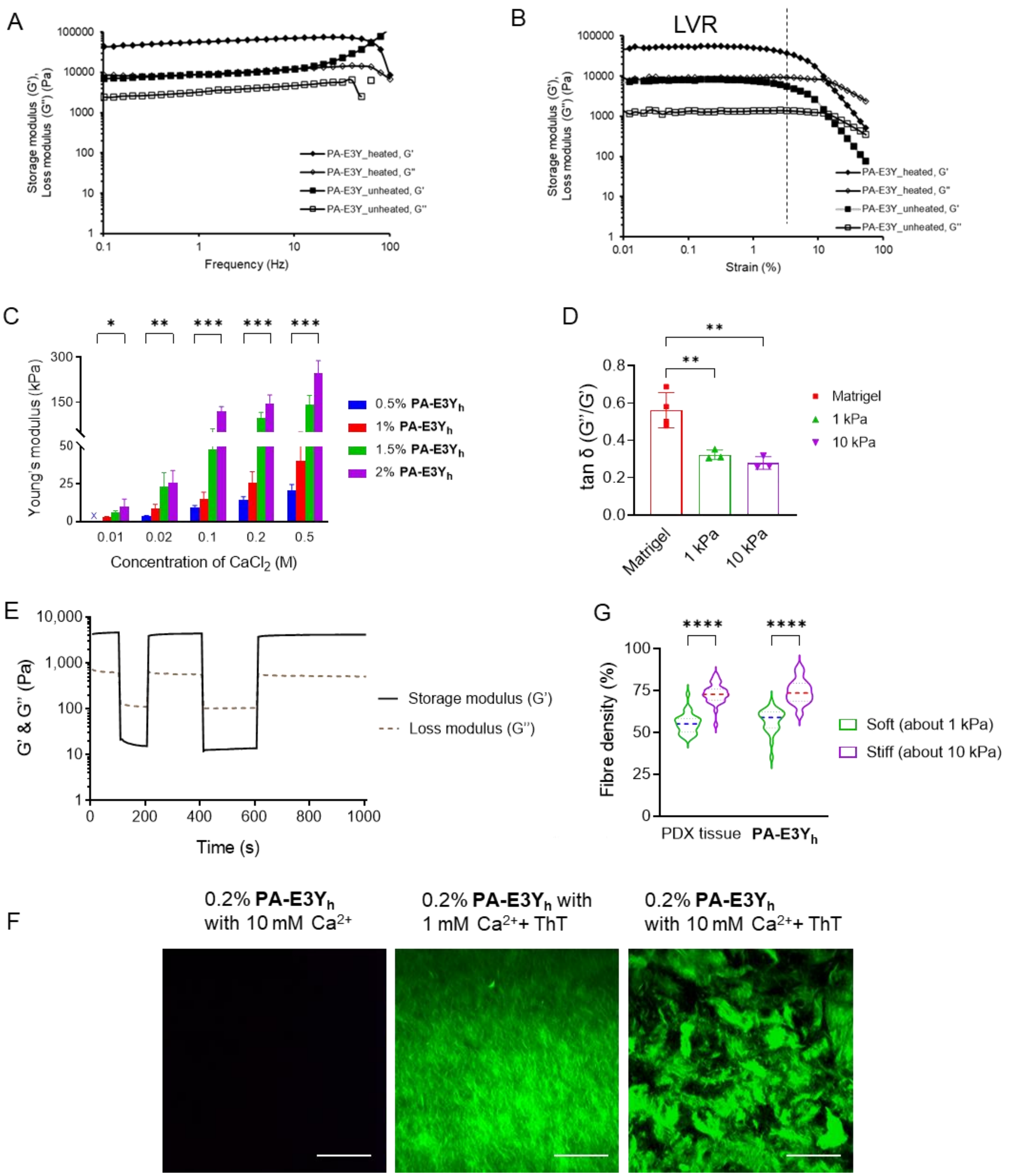

Figure S2. Characteristics of E3Y PA gel. (**A**) Frequency sweep rheographs of **PA-E3Y** and **PA-E3Y$_h$** hydrogels at 1.0 % wt/v gelator concentration. (**B**) Amplitude sweep rheographs of **PA-E3Y** and **PA-E3Y$_h$** hydrogels 1.0 % wt/v gelator concentration. (**C**) The stiffness of the **PA-E3Y$_h$** hydrogels increased as the gelator increased (n = 3). (**D**) Tan δ values of Matrigel, 1 kPa and 10 kPa **PA-E3Y$_h$** hydrogels. Tan δ (the ratio of G″/G′) represented the elasticity of the hydrogel, the lower Tan δ value, the more elastic the gel (n = 3). (**E**) Self-recovery or thixotropic property of 1%

PA-E3Y$_h$ hydrogels prepared with 0.05 M $CaCl_2$. Thixotropic behaviour of **PA-E3Y$_h$** hydrogel was measured at a constant frequency of 10 Hz and strain of 0.1% (100 s), 100% (100 s), 0.1% (200 s), 100% (200 s), and 0.1% (400 s). (**F**) Confocal micrographs of nanofibers of **PA-E3Y$_h$** with different concentrations of $CaCl_2$. Thioflavin T (0.4 mM) stained five times diluted 0.2% **PA-E3Y$_h$** treated with 1 mM and 10 mM of $CaCl_2$. The 0.2% **PA-E3Y$_h$** –Ca^{2+} at 10 mM without the ThT was used as a control, and there was no fluorescent signal detected (n = 3, scale bar: 100 μm). (**G**) Comparative analysis of the fiber density of PDX tissue and **PA-E3Y$_h$** hydrogels. * $P < 0.05$; ** $P < 0.01$; *** $P < 0.001$; **** $P < 0.0001$.

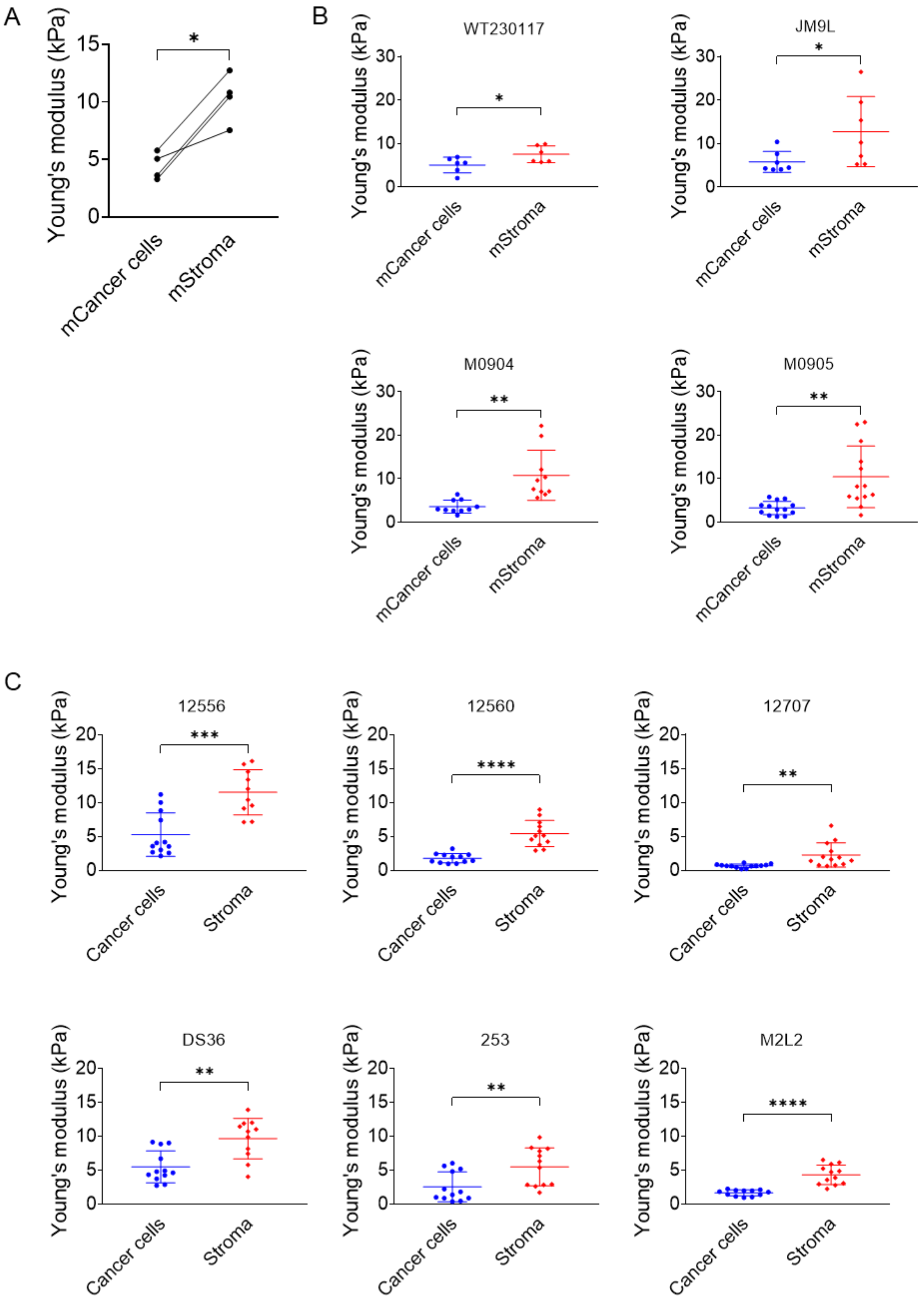
A
Young's modulus (kPa)
15
10
5
0
*
mCancer cells
mStroma
B
WT230117
JM9L
M0904
M0905
30
20
10
0
*
**
C
12556
12560
12707
DS36
253
M2L2
20
15
***
****
Cancer cells
Stroma

Figure S3. Stiffness of malignant and ECM area in pancreatic cancer patient-derived xenografts (PDXs) and mouse-derived allografts (MDAs). (**A**) Average stiffness of cancer cells and stroma areas in 4 MDAs tissue (n = 15). (**B**) Stiffness of cancer cells and stroma areas in 4 MDAs tissue (n = 15). (**C**) Stiffness of cancer cells and stroma areas in the 6 PDXs tissue (n = 15). Three frozen tissue sections for each specimen were independently measured by AFM. Five force maps were obtained from each section, and every force map covered a size of 50 × 50 μm^2 region under 10 × 10 point grids representing 100 force curves. * $P < 0.05$; ** $P < 0.01$; *** $P < 0.001$; **** $P < 0.0001$.

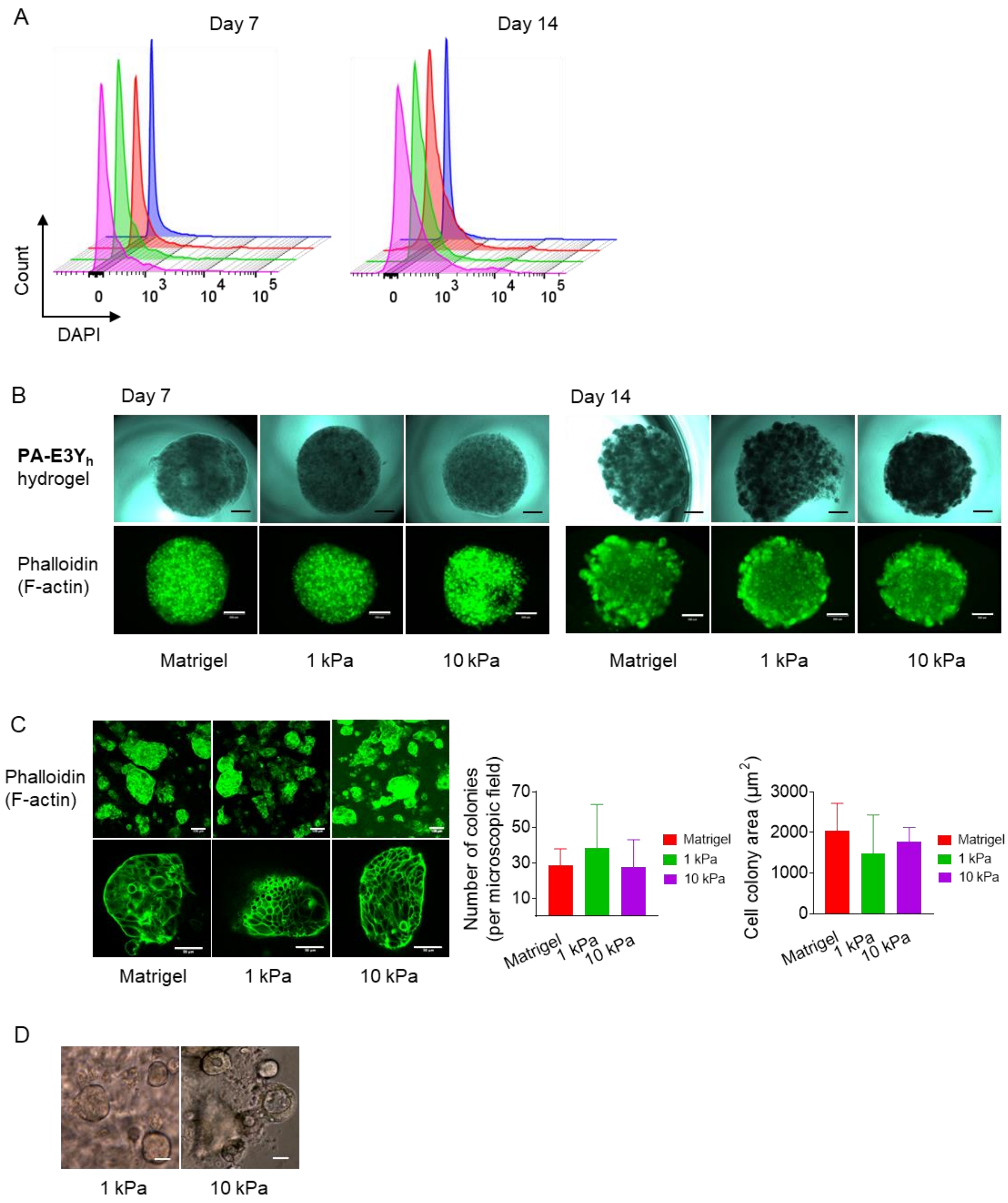

Figure S4. Cell viability and morphology of PDAC in Matrigel, 1 kPa and 10 kPa PA-E3Y$_h$ hydrogel. (**A**) Representative flow cytometry histograms for PDAC live cells (12556) derived from 2D, Matrigel, 1 and 10 kPa **PA-E3Y$_h$** hydrogels. (**B**) Images of PDAC cells seeded in Matrigel, 1 and 10 kPa **PA-E3Y$_h$** hydrogel for 7 and 14 days. Optical images of cell laden Matrigel and **PA-**

$E3Y_h$ hydrogels on Day 7 and 14 were taken on an optical microscope (Scale bar: 500 µm); and images of PDAC cells stained with Phalloidin in Matrigel and **PA-$E3Y_h$** hydrogels on Days 7 and 14 were taken by fluorescent microscopy (Scale bar: 500 µm. $n = 3$). (**C**) Cell colony formation in Matrigel, 1 and 10 kPa **PA-$E3Y_h$** hydrogels for 14 days. The images at left side: The cell morphology was stained by Phalloidin and the images were taken by confocal microscopy (Scale bar in the upper images: 100 µm; scale bar in the bottom images: 50 µm). The histogram at right side: Analysis of the size and number of cell colonies in Matrigel, 1 and 10 kPa **PA-$E3Y_h$** hydrogels ($n = 3$). (**D**) Optical images of duct-like structure of PDAC in 1 and 10 kPa **PA-$E3Y_h$** hydrogels (Scale bar: 100 µm, $n = 3$). * $P < 0.05$; ** $P < 0.01$; *** $P < 0.001$; **** $P < 0.0001$.

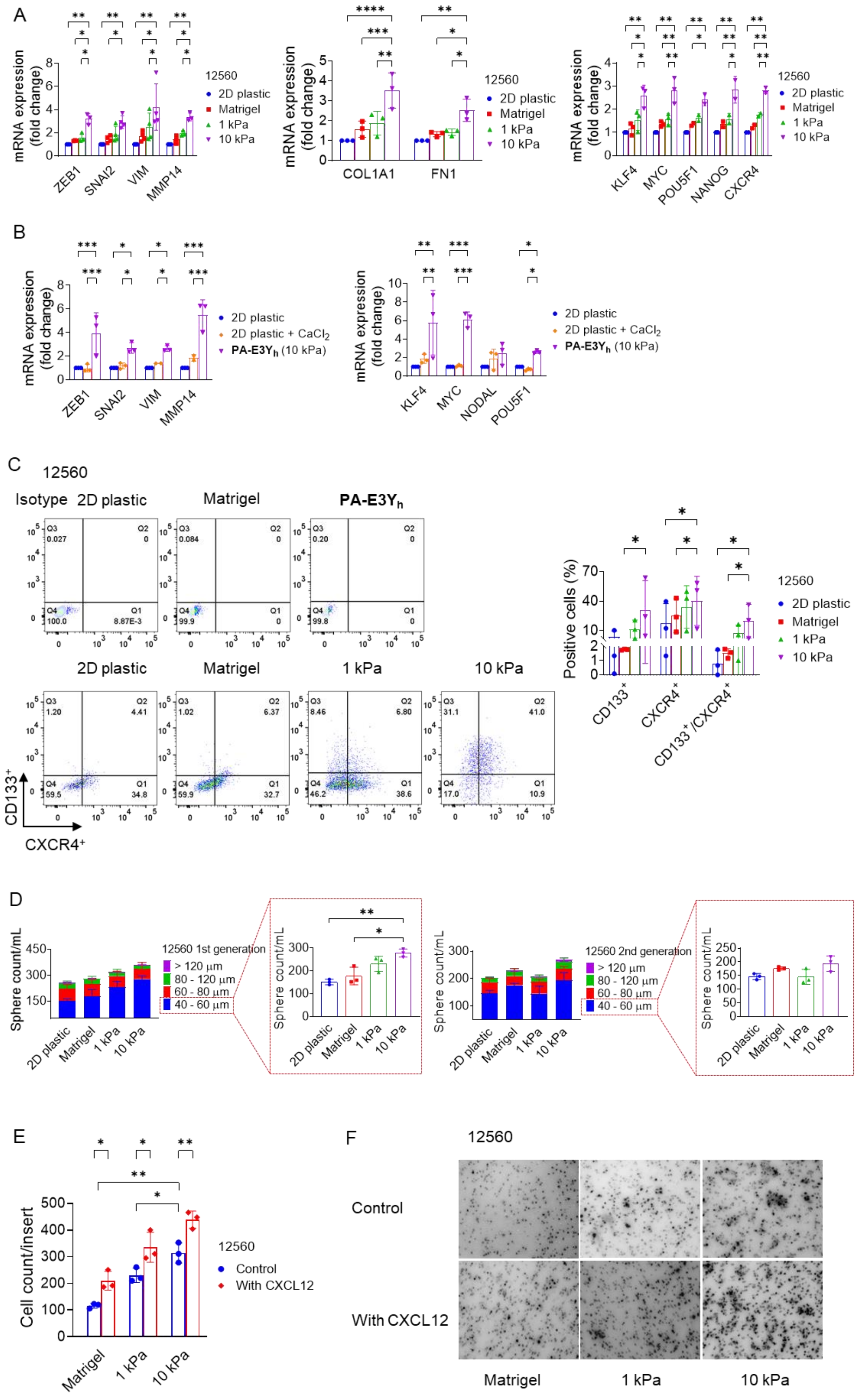
A
mRNA expression (fold change)
ZEB1
SNAI2
VIM
MMP14
COL1A1
FN1
KLF4
MYC
POU5F1
NANOG
CXCR4
12560
2D plastic
Matrigel
1 kPa
10 kPa
B
2D plastic + CaCl$_2$
PA-E3Y$_h$ (10 kPa)
NODAL
C
Isotype
PA-E3Y$_h$
Positive cells (%)
CD133$^+$
CXCR4$^+$
CD133$^+$/CXCR4$^+$
D
Sphere count/mL
12560 1st generation
12560 2nd generation
> 120 μm
80 - 120 μm
60 - 80 μm
40 - 60 μm
E
Cell count/insert
Control
With CXCL12
F

Figure S5. Matrix stiffness affects EMT and CSC phenotype in PDAC. (**A**) EMT, ECM and CSC related gene expression of PDAC cells (12560) cultured on 2D plastic, in Matrigel, 1, and 10 kPa **PA-E3Y$_h$** hydrogels on day 4 were investigated by qPCR (*n* = 3). (**B**) EMT and CSC related gene expression of PDAC cells on 2D plastic, 2D plastic with $CaCl_2$ and 10 kPa **PA-E3Y$_h$** hydrogel (*n* = 3). The 10 kPa **PA-E3Y$_h$** hydrogel culture: The 10 kPa stiffness of **PA-E3Y$_h$** hydrogels were prepared by mixing **PA-E3Y$_h$** and 50 mM $CaCl_2$, and the cell-laden hydrogel was cultured within 2 mL of sphere medium. 2D plastic with $CaCl_2$ culture: PDAC cells were cultured on 2D plastic within 2 mL of sphere medium plus the same amount of 50 mM $CaCl_2$ as 10 kPa **PA-E3Y$_h$** hydrogel. (**C**) $CD133^+/CXCR4^+$ CSC subpopulations were detected by flow cytometry for PDAC cells (12560) cultured in 2D plastic, Matrigel, 1 kPa and 10 kPa **PA-E3Y$_h$** hydrogels. Representative flow cytometry plots are shown on the left, and the analysis for the percentage of $CD133^+$, $CXCR4^+$ and $CD133^+/CXCR4^+$ populations is provided on the right side (*n* = 3). (**D**) Number of spheres by size range formed by PDAC cells (12560) derived from 2D plastic, Matrigel, 1 and 10 kPa **PA-E3Y$_h$** hydrogel for first and second generation spheres (*n* = 3). (**E**) Invasive capacity of PDAC cells (12560) derived from 2D plastic, Matrigel, 1 and 10 kPa **PA-E3Y$_h$** hydrogels as assayed in Matrigel-coated transwell inserts, in the absence or presence of the specific chemoattractant CXCL12 (*n* = 3). (**F**) Representative images of invasive PDAC cells (12560) derived from 2D plastic, Matrigel and **PA-E3Y$_h$** hydrogel, transferred through the Matrigel-coated inserts with and without CXCL12. * $P < 0.05$; ** $P < 0.01$; *** $P < 0.001$; **** $P < 0.0001$.

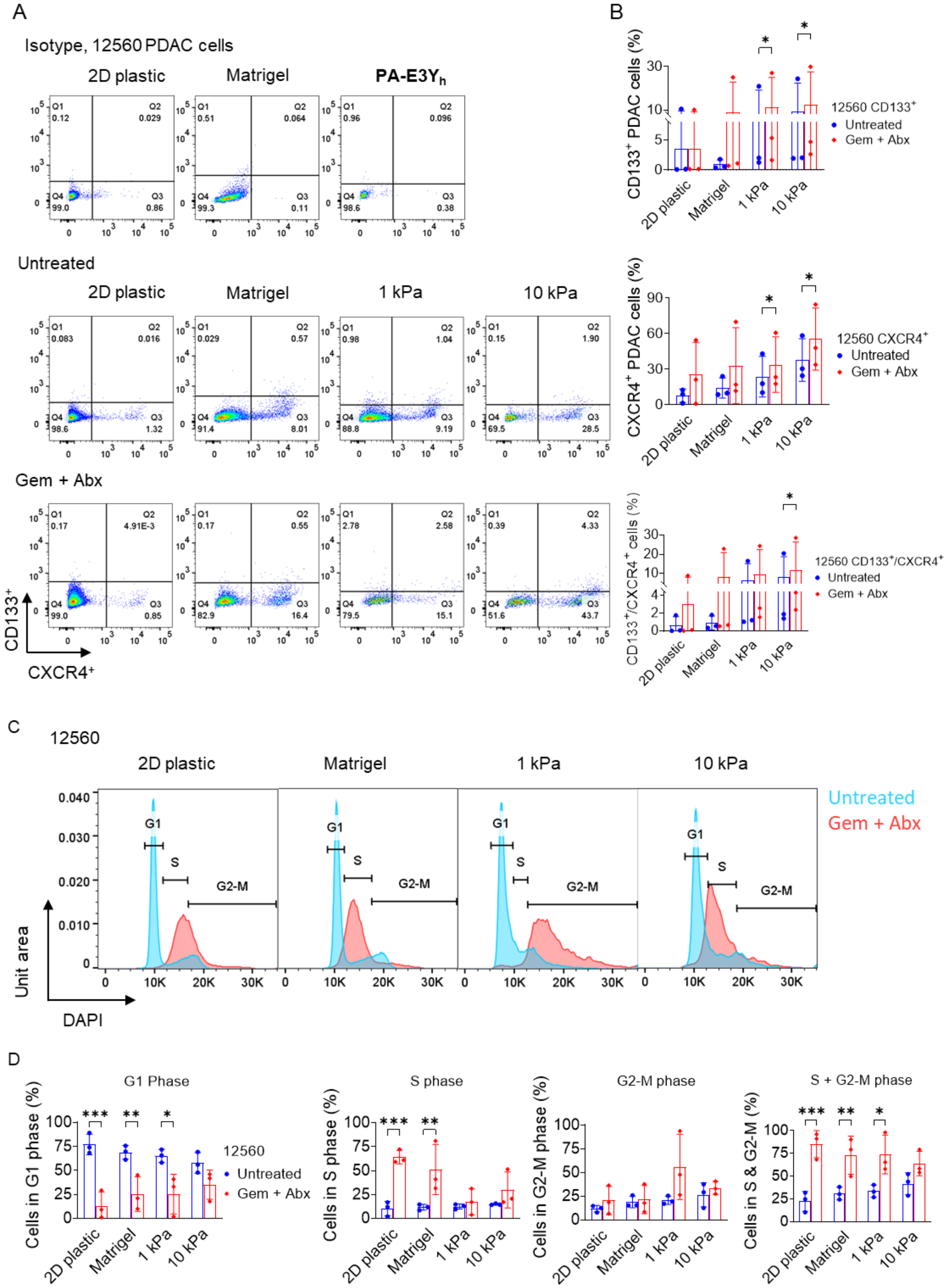
A
Isotype, 12560 PDAC cells
2D plastic
Matrigel
PA-E3Yh
Untreated
1 kPa
10 kPa
Gem + Abx
CD133+
CXCR4+
B
CD133+ PDAC cells (%)
12560 CD133+
Untreated
Gem + Abx
CXCR4+ PDAC cells (%)
12560 CXCR4+
CD133+/CXCR4+ cells (%)
12560 CD133+/CXCR4+
C
12560
Untreated
Gem + Abx
G1
S
G2-M
Unit area
DAPI
D
G1 Phase
S phase
G2-M phase
S + G2-M phase
Cells in G1 phase (%)
Cells in S phase (%)
Cells in G2-M phase (%)
Cells in S & G2-M (%)

Figure S6. Matrix stiffness affects enrichment of $CD133^{+}/CXCR4^{+}$ CSC population during chemotherapy. (**A,B**) $CD133^{+}/CXCR4^{+}$ CSC population during drug treatment as evaluated by flow cytometry (*n* = 3). (**A**) Representative flow cytometry plots for $CD133^{+}$, $CXCR4^{+}$, and $CD133^{+}/CXCR4^{+}$ CSC subgroups in PDAC cells (12560) in the absence and presence of drug treatment. (**B**) Analysis of $CD133^{+}$, $CXCR4^{+}$ and $CD133^{+}/CXCR4^{+}$ CSC populations (*n* = 3). (**C,D**) Response of PDAC cell cycle to Gem/Abx in 2D, Matrigel, 1 and 10 kPa **PA-E3Y$_h$** hydrogels. (**C**) The representative cell cycle profiles of untreated (blue) and treated (red) PDAC cells (12560) gated to exclude debris and doublets. (**D**) Percentages represent the proportion of PDAC cells (12560) that were in G1, S, G2-M and S+G2-M phases compared to untreated controls (*n* = 3). * $P < 0.05$; ** $P < 0.01$; *** $P < 0.001$; **** $P < 0.0001$.

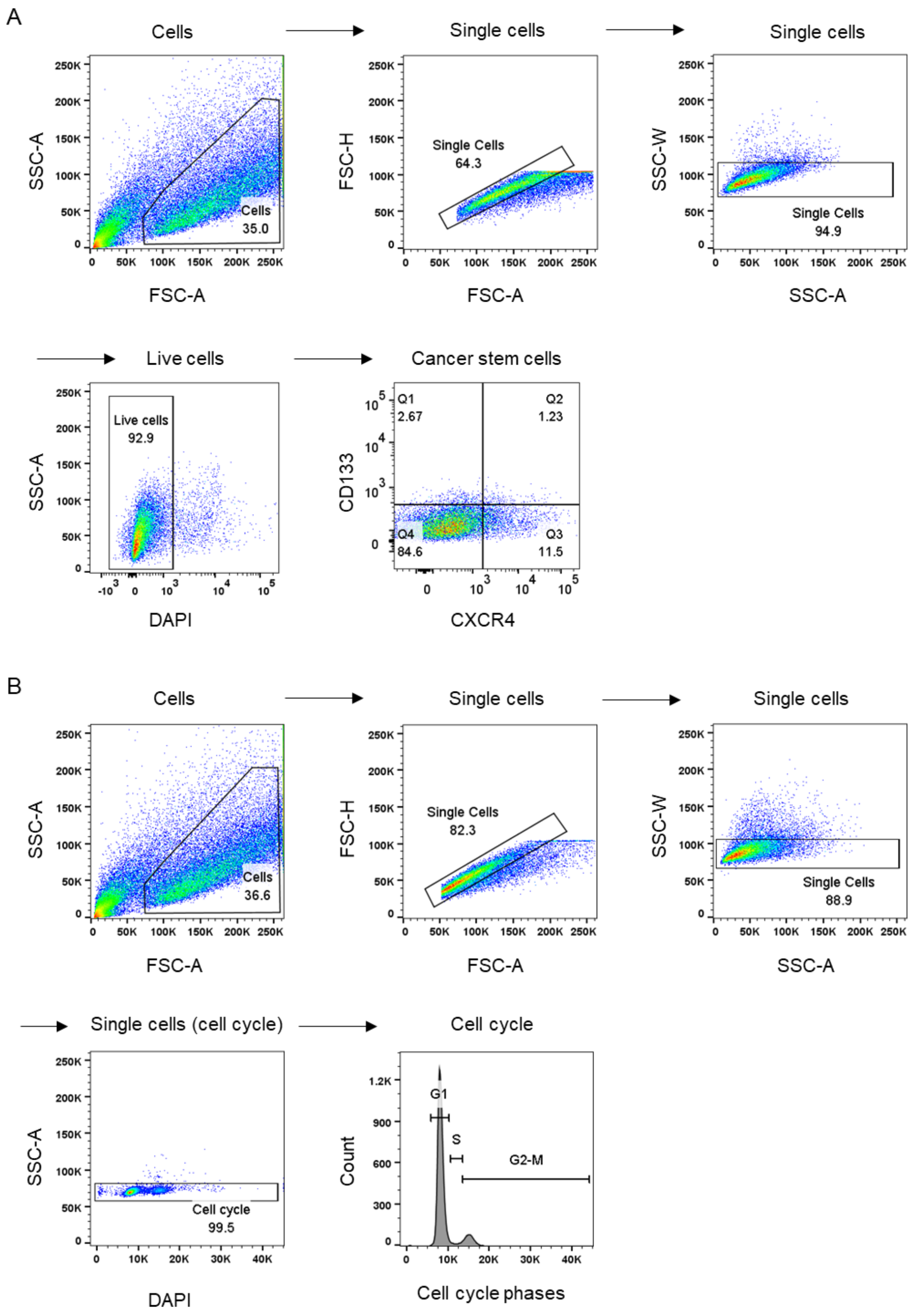

Figure S7. Flow cytometry gating strategy. (**A**) Flow cytometry gating strategy of CSC marker analysis. (**B**) Flow cytometry gating strategy of cell cycle analysis after drug treatment. SSC = side scatter, FSC = forward scatter.

The **order** of sample in WB: 2D plastic, Matrigel, 1 kPa, 10 kPa.

Membrane 1

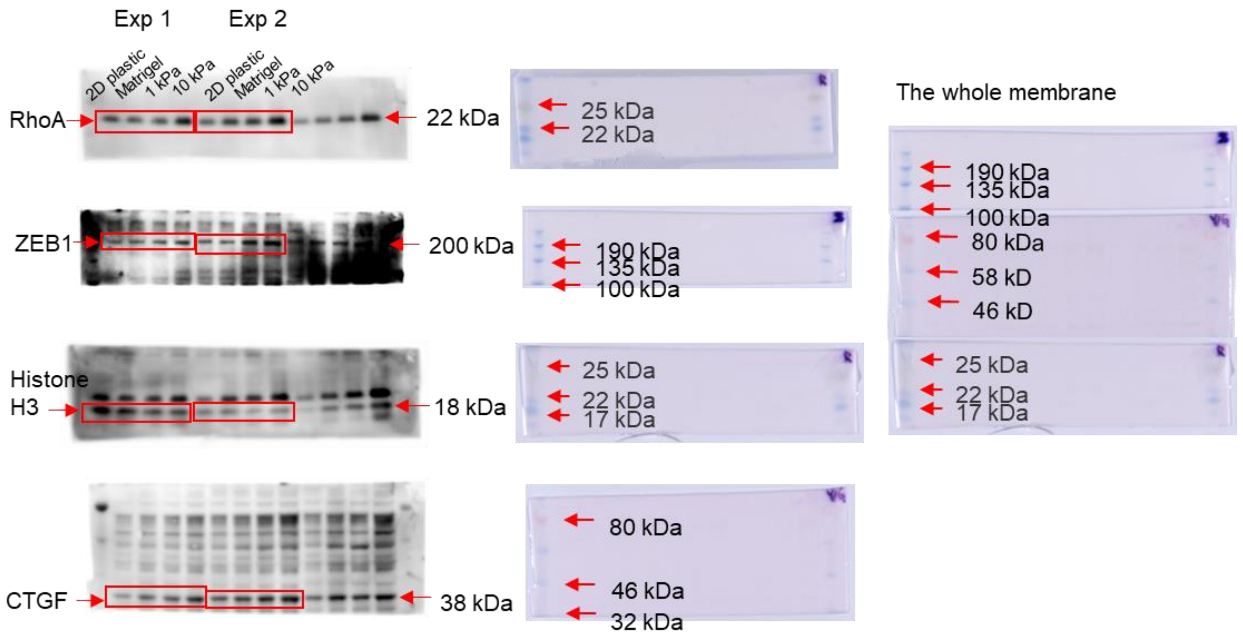

Membrane 2

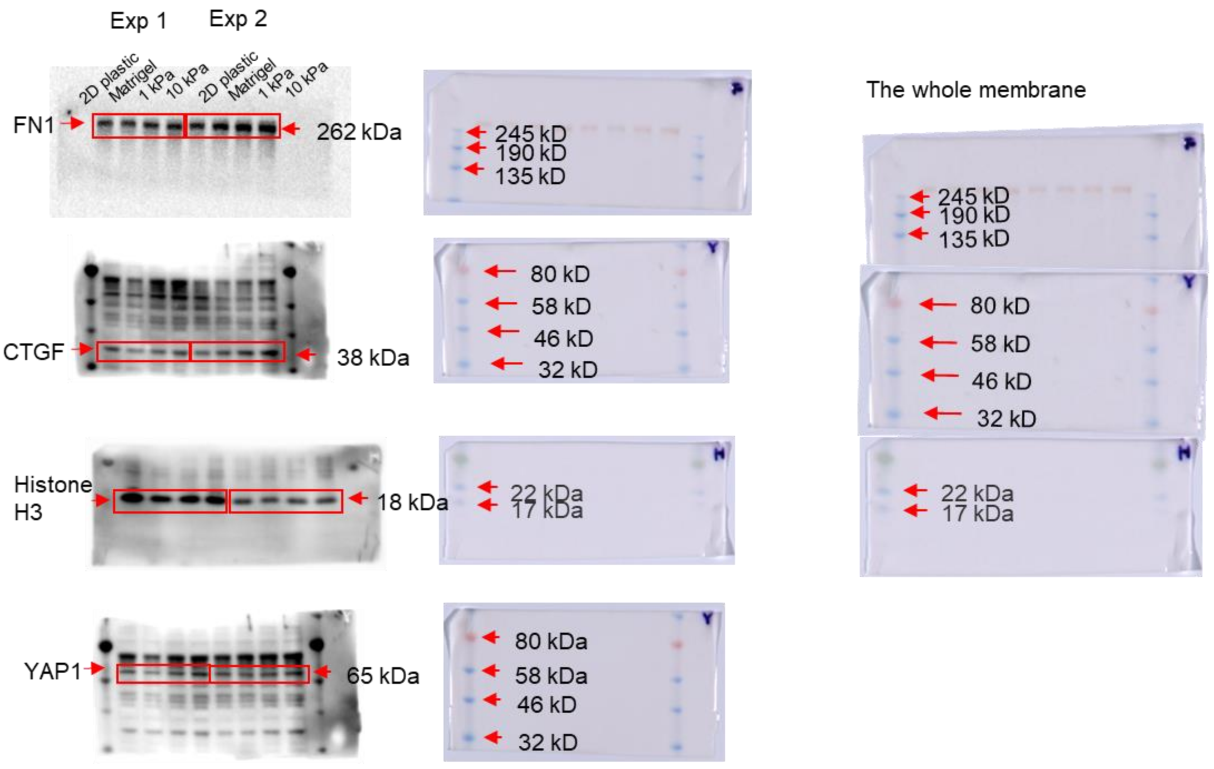

The **order** of sample in WB: 2D plastic, Matrigel, 1 kPa, 10 kPa.

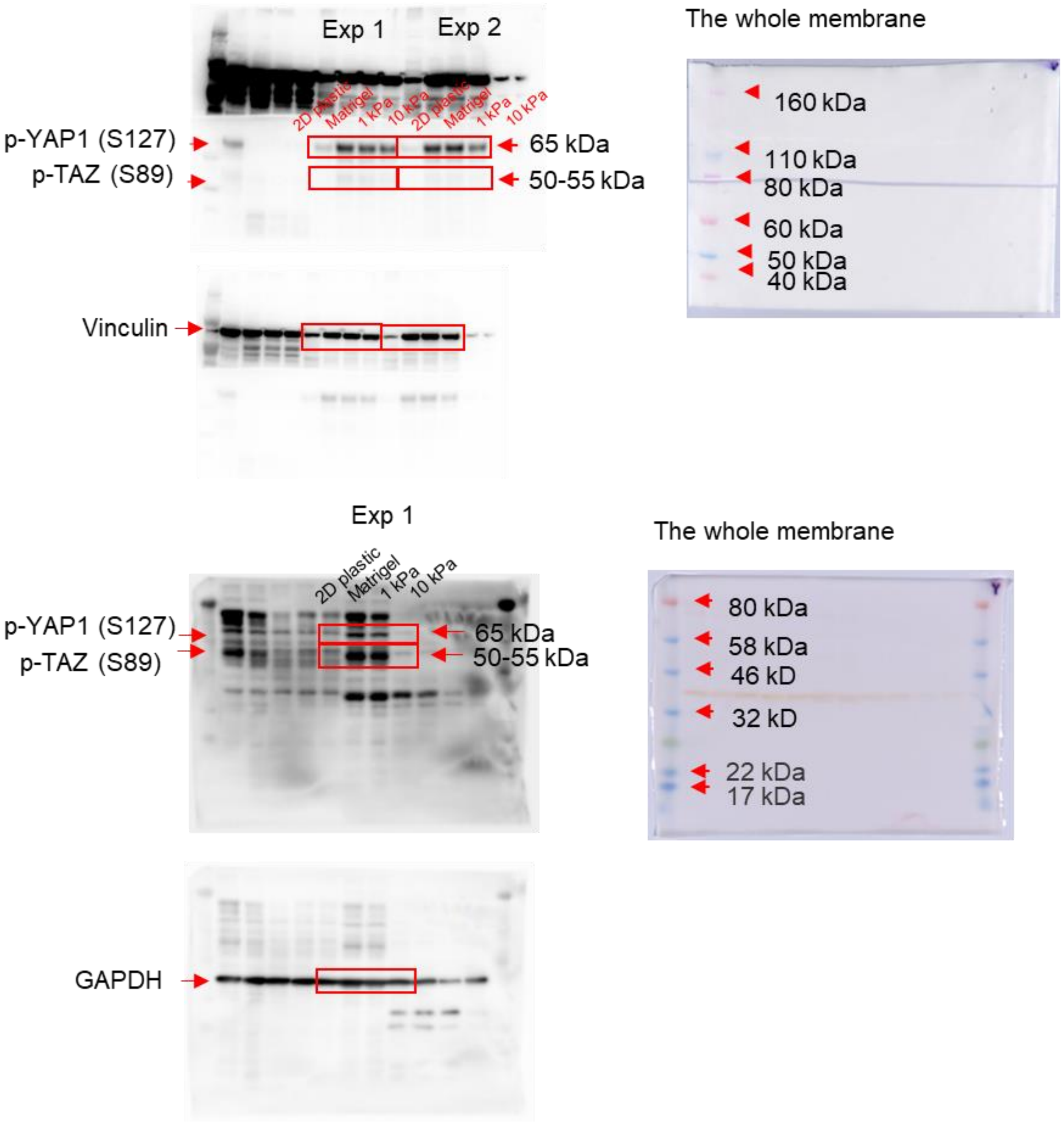

Figure S8. Raw data of western blot.
